# Finding distributions that differ, with false discovery rate control

**DOI:** 10.1093/biomet/asag025

**Published:** 2026-04-04

**Authors:** Yonghoon Lee, Edgar Dobriban, Eric J Tchetgen Tchetgen

**Affiliations:** Department of Statistics and Data Science, The Wharton School, University of Pennsylvania, 265 South 37th Street, Philadelphia, Pennsylvania 19104, U.S.A; Department of Statistics and Data Science, The Wharton School, University of Pennsylvania, 265 South 37th Street, Philadelphia, Pennsylvania 19104, U.S.A; Department of Statistics and Data Science, The Wharton School, University of Pennsylvania, 265 South 37th Street, Philadelphia, Pennsylvania 19104, U.S.A

**Keywords:** Distribution-free inference, False discovery rate, Multiple testing

## Abstract

We consider the problem of comparing a reference distribution with several other distributions. Given a sample from both the reference and the comparison groups, we aim to identify the comparison groups whose distributions differ from that of the reference group. Viewing this as a multiple-testing problem, we introduce a methodology that provides exact, distribution-free control of the false discovery rate. To do so, we introduce the concept of *batch conformal p -values* and demonstrate that they satisfy positive regression dependence across the groups Benjamini & Yekutieli (2001), thereby enabling control of the false discovery rate through the Benjamini–Hochberg procedure. The proof of positive regression dependence introduces a novel technique for the inductive construction of rank vectors with almost-sure dominance under exchangeability. We evaluate the performance of the proposed procedure through simulations. Despite being distribution-free, in some cases it shows performance comparable to methods with knowledge of the data-generating normal distribution, and it further has more power than direct approaches based on conformal out-of-distribution detection. Furthermore, we illustrate our methods on a hepatitis C treatment dataset, where they identify patient groups with large treatment effects, and on the Current Population Survey dataset, where they identify subpopulations with long working hours.

## 1. Introduction

### 1.1. Overview

We consider the problem of identifying groups whose distributions differ from a given reference distribution. Such comparisons arise frequently, for instance when evaluating whether a factor such as a treatment impacts a medical outcome compared to a control condition. Formally, given data sampled from a reference distribution $ P $ and comparison distributions $ P^{(1)},\ldots,P^{(K)} $, we consider testing the $ K $ null hypotheses $ H_{k}\colon P^{(k)}=P,\,k=1,2,\ldots,K $. For instance, in evaluating the treatment outcome $ T $ in its association with an outcome variable $ X $, we may compare the distributions $ P_{X\mid T=t_{k}} $ of the outcome given treatment level $ t_{k} $ with the distribution $ P_{X\mid T=t_{0}} $ of the outcome under the control condition $ t_{0} $, corresponding to the null hypothesis $ H_{k}\colon P_{X\mid T=t_{0}}=P_{X\mid T=t_{k}} $.

The global null, under which all null hypotheses $ H_{k},\,k=1,2,\ldots,K $, hold, corresponds to the hypothesis of independence, $ H_{0}\colon T \indep X $. This has been widely studied, with classical methods such as permutation tests providing exact Type I error control without requiring explicit distributional assumptions (Eden & Yates, 1933; Fisher, 1935; Kennedy, 1995; Anderson, 2001; Pesarin, 2001; Ernst, 2004; Good, 2006; Pesarin & Salmaso, 2010b, 2012; Hemerik & Goeman, 2018).

However, these methods are only informative about the global null, i.e., whether the factor has any influence. In practice, researchers often require more nuanced insights, specifically identifying which particular groups (or treatment levels) deviate significantly from the reference. This problem can be formulated as a multiple-testing problem (see, e.g., Shaffer, 1995; Lehmann & Romano, 2022), in which one simultaneously tests hypotheses $ H_{k}\colon P_{X\mid T=t_{0}}=P_{X\mid T=t_{k}} $. Ensuring reliable inference in this multiple-testing setting requires control of error measures such as the false discovery rate (Benjamini & Hochberg, 1995), ideally under minimal distributional assumptions.

A special case of crucial importance is when there is only one comparison distribution: this corresponds to the two-sample testing problem, where we test $ P^{(1)}=P $ (see, e.g.,Pitman, 1937; Pesarin & Salmaso, 2010a, 2013; Pesarin, 2015; Kim et al., 2020, 2022; Lehmann & Romano, 2022). However, addressing multiple comparisons poses unique challenges. In particular, dependence among test statistics arising from the shared reference observations poses a challenge to the application and construction of valid and powerful multiple-testing procedures.

We address this challenge by proposing a novel distribution-free method for controlling the false discovery rate in the multiple-group comparison setting. Our contributions can be summarized as follows.

(i) *Batch conformal $ p $-values for distribution-free testing*. We introduce the concept of *batch conformal $ p $-values*, an adaptation of batch predictive inference (Lee et al., 2025), to enable rigorous distribution-free tests for equality of distributions between datasets. Specifically, the batch conformal $ p $-values reject the null hypothesis when the quantiles of the distributions being compared differ.
(ii) *Multiple testing with exact false discovery rate control via positive regression dependence*. We establish a theoretical result demonstrating that batch conformal $ p $-values exhibit positive regression dependence across groups (Benjamini & Yekutieli, 2001). This property justifies the application of the Benjamini–Hochberg procedure (Benjamini & Hochberg, 1995) for exact control of the false discovery rate when testing for distributional shifts across multiple groups. The cornerstone of our proof is a novel inductive method for constructing rank vectors that exhibit almost-sure dominance under exchangeability.
(iii) *A conformal-type procedure for simultaneous testing of equality of distributions*. Our procedure enjoys the advantages of general conformal-type methods: it is simple and computationally efficient, and provides exact finite-sample false discovery rate (FDR) control in a distribution-free sense. The rejection rule can be tailored to the target of interest through an appropriate score function chosen by the user, and any machine-learning algorithm can be used to construct the score, for arbitrary sample sizes.
(iv) *Empirical validation demonstrating near-oracle performance*. Through simulations, we demonstrate that our distribution-free approach controls the false discovery rate while often achieving power comparable to model-based methods under correctly specified parametric settings. At the same time, it significantly outperforms direct conformal methods designed for out-of-distribution detection (Bates et al., 2023).
(v) *Illustrations on empirical datasets*. We illustrate our methods using two datasets: the HALT-C study on hepatitis C treatment effects (Snow, 2024) and the CurrentPopulation Survey (CPS; U.S. Census Bureau, 2024). Specifically, we identify patient groups with significant treatment responses in the HALT-C dataset and pinpoint subpopulations exhibiting notably longer working hours in the CPS dataset.

Notation in this paper is as follows. We denote the set of real numbers by $ \mathbb{R} $. For a set $ \mathcal{X} $, $ \mathcal{X}^{n} $ denotes its $ n $-dimensional product space. For a positive integer $ n $, we write $ [n] $ to denote the set $ \{1,2,\ldots,n\} $ and $ \mathcal{S}_{n} $ to denote the set of permutations $ \{\sigma\colon[n]\rightarrow[n],\sigma\text{ is a bijection}\} $. For a distribution $ P $ and $ \tau\in(0,1) $, the quantile function $ Q_{\tau}(P)=\inf\{t\in\mathbb{R}\colon\mathbb{P}_{{X\sim Q}}\left\{{X\leqslant t }\right\}\geqslant\tau\} $ denotes the $ \tau $th quantile of $ P $, where the infimum is defined as infinity if the set is empty. If a finite set or multiset $ A\subset\mathbb{R} $ is provided as input to $ Q_{\tau} $, then $ Q_{\tau}(A) $ represents the quantile of the uniform distribution over $ A $. The indicator of an event $ E $ is denoted by $ {\mathbb{1}}\{{E}\} $. For vectors $ u,v\in\mathbb{R}^{d} $, we write $ u\preceq v $ if $ u_{i}\leqslant v_{i} $ for all $ i\in[d] $.

### 1.2. Problem set-up

**Figure 1:**
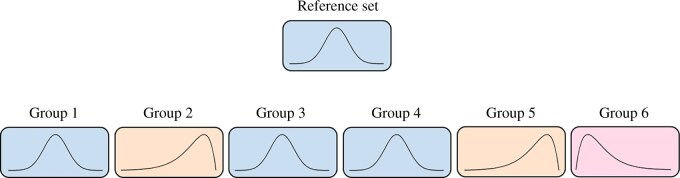
Visual representation of the problem set-up. In this example, the objective is to identify correctly the comparison groups (groups 2, 5 and 6) whose distributions differ from that of the reference set, using data points from each group.

We consider a setting in which our observations are drawn from either a reference distribution or several comparison distributions. Specifically, we observe a*reference dataset* $ (X_{1},\ldots,X_{n})\subset\mathcal{X}^{n} $ drawn independently and identically distributed from a reference distribution $ P $. Moreover, we observe $ K $ independent *comparison datasets* $ G_{1},\ldots,G_{K} $, where each $ \smash{G_{k}=(X_{1}^{(k)},X_{2}^{(k)},\ldots,X_{n_{k}}^{(k)})\subset\mathcal{X }^{n_{k}}} $ is drawn independently and identically distributed from a comparison distribution $ P^{(k)} $. We are interested in selecting groups $ k $ whose comparison distribution $ \smash{P^{(k)}} $ differs from the reference distribution. An illustration of the problem set-up is given in Fig. 1. Specifically, we consider the multiple-testing problem with the hypotheses


(1) \begin{align*} H_{k}\colon P^{(k)}=P,\qquad k=1,2,\ldots,K. \end{align*}

As usual in multiple hypothesis testing (Lehmann & Romano, 2022), our goal is to use the data to select a subset $ S\subset\{1,\ldots,K\} $ that captures most of the indices $ k $ for which $ P^{(k)}\not= P $. At the same time, we aim to control the number of selected indices $ k $ for which $ P^{(k)}=P $, e.g., where a treatment of interest has no effect.

### 1.3. Related work

The related literature is too vast to summarize here; therefore, we review only the most relevant prior works. Testing the equality of distributions has been investigated in various contexts, ranging from parametric methods, such as the two-sample $ t $-test (Student, 1908), to nonparametric approaches, including the Kolmogorov–Smirnov test (Kolmogorov, 1933; Smirnov, 1948), the energy distance method (Székely & Rizzo, 2004) and the maximum mean discrepancy test (Gretton et al., 2012). Nonparametric approaches with weak assumptions have also been studied; see, for example, recent developments by Kim et al. (2020), Lin et al. (2023), Hu & Lei (2024) and Hu & Lin (2025). Additionally, distribution-free tests, such as the permutation test (Eden & Yates, 1933; Fisher, 1935; Kennedy, 1995; Anderson, 2001; Pesarin, 2001; Ernst, 2004; Good, 2006; Pesarin & Salmaso, 2010b, 2012; Hemerik & Goeman, 2018) and the rank-sum test (Mann & Whitney, 1947), have been widely studied. For more recent developments, see Rosenbaum (2005) and Biswas et al. (2014).

While the aforementioned studies primarily focus on comparing two distributions, our work addresses the multiple-testing problem in which multiple comparison distributions are compared against a reference distribution. Similar problems have been explored in several works, starting with the Dunnett procedure (Dunnett, 1955) for normally distributed data. Hothorn (2020) provided a simulation study comparing the Dunnett $ t $-test with closed testing procedures. In the distribution-free inference literature, such many-to-one comparisons have been extensively explored in the context of outlier or novelty detection. Bates et al. (2023) presented a strategy for using conformal $ p $-values to detect outliers while controlling the false discovery rate in a distribution-free setting. Their method was extended in Magnani et al. (2024) and Marandon et al. (2024). Additionally, Lee et al. (2025) proposed a methodology based on full conformal $ e $-values (Vovk & Wang, 2021), enabling more efficient use of the data.

Controlling the false discovery rate in multiple-testing problems has been a topic of significant interest, starting with the Benjamini–Hochberg procedure introduced by Benjamini & Hochberg (1995). Benjamini et al. (2006) proposed an extension of the procedure with tighter false discovery rate control. Several subsequent works have studied conditions beyond independence of the input $ p $-values under which the Benjamini–Hochberg procedure achieves valid false discovery rate control. Benjamini & Yekutieli (2001) showed that this holds under the more general condition of positive regression dependence that they introduced. The theoretical properties under positive regression dependence were further studied by Sarkar (2002). Blanchard & Roquain (2008) studied conditions for false discovery rate control with extensions of the Benjamini–Hochberg procedure; see Benjamini (2010) for a review.

## 2. Methodology and theoretical results

### 2.1. Review of the Benjamini–Hochberg procedure

In the setting of multiple hypothesis testing, the Benjamini–Hochberg procedure (Benjamini & Hochberg, 1995) is a widely used methodology. Given a target level $ \alpha\in(0,1) $ and $ p $-values $ p_{1},\dots,p_{M} $ for hypotheses $ H_{1},\dots,H_{M} $, the Benjamini–Hochberg procedure computes $ k^{*}=\max\{k\colon p_{(k)}\leqslant{k}\alpha/{M}\} $, where $ p_{(k)} $ denotes the $ k $th order statistic of the $ p $-values. It then rejects all null hypotheses for which the corresponding $ p $-value is less than or equal to $ p_{(k^{*})} $.

The false discovery rate is defined as $ \mathbb{E}\left[{V/\max(R,1)}\right] $, where $ V $ is the number of false discoveries, i.e., the number of rejected hypotheses that are true or null, and $ R $ is thenumber of rejected hypotheses. The Benjamini–Hochberg procedure is known to control the false discovery rate at the predefined level $ \alpha $ when the $ p $-values $ p_{1},\dots,p_{M} $ are independent (Benjamini & Hochberg, 1995), or when they satisfy the *positive regression dependence on a subset* (PRDS) property on the set of true null hypotheses (Benjamini & Yekutieli, 2001).

To define this property, we first introduce the following definitions. For a positive integer $ d $ and two vectors $ v=(v_{1},\ldots,v_{d})^{\mathrm{\scriptscriptstyle T}} $ and $ w=(w_{1},\ldots,w_{d})^{\mathrm{\scriptscriptstyle T}} $ in $ \mathbb{R}^{d} $, we write $ v\preceq w $ if $ v_{j}\leqslant w_{j} $ for all $ j\in[d] $. A set $ A\subset\mathbb{R}^{d} $ is called increasing if, for any $ x\in A $, $ x\preceq y $ implies that $ y\in A $.

Definition 1. (Benjamini & Yekutieli, 2001). *For a set $ I\subset\{1,\ldots,K\} $, a random vector $ X=(X_{1},X_{2},\ldots,X_{K}) $ is positive regression dependent on $ I $ if, for all $ k\in I $, the conditional probability $ x\mapsto\mathbb{P}\left\{{X\in A}\ \middle|\ {X_{k}=x}\right\} $ is nondecreasing on its domain as a function of $ x\in\mathbb{R} $ for any increasing set $ A\subset\mathbb{R}^{K} $.*

Benjamini & Yekutieli (2001) showed that if the $ p $-value vector $ (p_{1},\ldots,p_{M})^{\mathrm{\scriptscriptstyle T}} $ is positive regression dependent on the set of nulls $ I_{0}=\{k\colon H_{k}\text{ is true}\} $, then the Benjamini–Hochberg procedure controls the false discovery rate at the desired level $ \alpha $, i.e., $ \mathbb{E}\left[{V/\max(R,1)}\right]\leqslant\alpha $.

### 2.2. Existing methods

In this section, we discuss several existing, i.e., baseline, approaches that one may consider for constructing $ p $-values for our selection problem, and illustrate their limitations.

1. *Partitioning the reference dataset*. As discussed in the previous section, one option for controlling the false discovery rate is to construct independent $ p $-values for $ H_{1},\dots,H_{K} $, and then apply the Benjamini–Hochberg procedure. The methods discussed in § 1 of the Supplementary Material can be used to construct a $ p $-value for an individual hypothesis. However, data points should be used only once, in order to ensure independence of the $ p $-values. To achieve this, one could split the reference dataset into $ K $ subsets and use each subset to construct a $ p $-value for each hypothesis $ H_{k},\,k\in[K] $, from (1). For example, if $ n=Kq+r $ for some positive integer $ q $ and $ 0\leqslant r< q $, then one can use the dataset $ \{X_{(k-1)q+1},X_{(k-1)q+2},\dots,X_{(k-1)q+q-1}\} $ to construct a permutation-test $ p $-value for $ H_{k} $.

However, this approach becomes problematic when the reference sample size is small. In that case, splitting the reference data into $ K $ subsets leads to very small datasets which in turn leads to inaccurate $ p $-values with low power. To handle this setting, it is desired to develop methods that permit reusing the reference data.

2. *Testing with conformal $ p $-values after subsampling*. An approach that allows reuse of the reference data is based on conformal testing for outliers, which has appeared in Bates et al. (2023) as part of a broader effort to construct calibration-set-conditional $ p $-values for outlier detection; see also Mary & Roquain (2022).

To introduce this, let $ s\colon\mathcal{X}\rightarrow\mathbb{R} $ be a *score* function that maps each observation to a real value and is constructed independently of the data. In practice, it is standard in related problems in conformal prediction to split the data into two subsets, using one to construct the score function and the other as the reference (calibration) dataset (Vovk et al., 2005). We choose a score such that larger scores are more likely to occur under $ \smash{P^{(k)}} $ when $ P \not= P^{(k)} $. Let $ S_{i},\,i\in[n] $, and $ \smash{S_{i}^{(k)}} $, $ k\in[K] $, $ i\in[n_{k}] $, denote the reference scores and the comparison scores of the $ k $th comparison group, respectively.

To use conformal testing for outliers (Bates et al., 2023), we randomly choose one data point from each $ G_{k} $ and apply the Benjamini–Hochberg procedure to the following $ p $-values:


\begin{align*} p_{k}=\frac{\sum\nolimits_{i=1}^{n}\mathbf{1}\{S_{i_{k}^{*}}^{(k)}\leqslant S_{i}\}+1}{n+1},\quad\mathrm{where}\quad i_{k}^{*}\sim{\rm Un}(\{1,2,\ldots,n_{k}\}). \end{align*}

Bates et al. (2023) considered detecting outliers, so they used one data point from each distribution, which in our context implies that their method requires subsampling. Bates et al. (2023) showed that the above subsampling conformal $ p $-values satisfy positive regression dependence, so that the Benjamini–Hochberg procedure ensures false discovery rate control.

However, this procedure discards most of the information from the comparison data, relying only on one randomly selected data point from each group. In our experiments, we show that this can lead to low power compared to our proposed method. Moreover, this line of work also provides another interpretation and possible application of our method to *batch outlier detection*, where one has batched observations and uses our method to detect ‘outlier batches’.

3. *Applying the Benjamini–Yekutieli procedure*. Alternatively, one may consider applying the Benjamini–Yekutieli procedure (Benjamini & Yekutieli, 2001) instead of the Benjamini–Hochberg procedure to any two-sample $ p $-values. The Benjamini–Yekutieli procedure applies the Benjamini–Hochberg procedure at a more conservative level $ \smash{\alpha/\sum_{i=1}^{K}(1/i)} $ and provides valid false discovery control under arbitrarily dependent $ p $-values. However, this procedure typically produces conservative results; therefore, we explore a way to construct $ p $-values that allow the use of the Benjamini–Hochberg procedure.

Remark 1. In addition to the approaches above that provide distribution-free finite-sample FDR control, one may also consider methods without theoretical guarantees; for example, applying the Benjamini–Hochberg procedure with $ p $-values constructed from existing two-sample testing methods. In § 3.2 below, we present experimental results for such approaches for comparison, using well-known $ p $-values from Gretton et al. (2012) and Ramdas et al. (2017).

### 2.3. Proposed method: testing with batch conformal $ p $-values

We now present our main procedure, which allows the repeated use of reference data. Consider a score function $ s\colon\mathcal{X}\rightarrow\mathbb{R} $ constructed in advance, independently of the data used for inference; see Vovk et al. (2005) and Angelopoulos & Bates (2022) for standard examples. For example, one can construct an estimated mean function $ \hat{\mu}(\cdot) $ using a split of the reference data, and then work with the residual score $ s\colon(x,y)\mapsto|y-\hat{\mu}(x)| $. If we are interested in comparing the outcomes themselves, we can choose $ s\colon(x,y)\mapsto y $. Furthermore, for each $ k\in[K] $, select an integer $ \eta_{k}\in[n_{k}] $ such that the primary interest lies in the $ \eta_{k}/n_{k} $th quantile of the distribution of the scores. We then define the batch conformal $ p $-value for $ H_{k} $ as


(2) \begin{align*} p_{k} &=\sum\limits_{i=1}^{n}\bigg\{{\binom{i+\eta_{k}-2}{\eta_{k}-1}\binom{n +n_{k}-i-\eta_{k}+1}{n_{k}-\eta_{k}}}\bigg{/}{\binom{n+n_{k}}{n_{k}}}\bigg\} \cdot{\mathbb{1}}\{{S_{(\eta_{k})}^{(k)}\leqslant S_{(i)}}\} \nonumber\\ &\quad+{\binom{n+\eta_{k}-1}{\eta_{k}-1}}\bigg{/}{\binom{n+n_{k}}{ n_{k}}}. \end{align*}

The $ i $th term in this expression computes the probability that the $ \eta_{k} $th smallest test score among a random size-$ n_{k} $ subset drawn from a combined population of $ n+n_{k} $ scores (consisting of both calibration and test points) is at least as large as the $ i $th smallest calibration score (e.g., Wilks, 1962, p. 243). The first term sums over all $ i\in[n] $, weighting by the probability that the $ \eta_{k} $th order statistic lands atposition $ i $, and then checking whether the observed test statistic $ \smash{S_{(\eta_{k})}^{(k)}\leqslant S_{(i)}} $. The final term corresponds to the probability that the $ \eta_{k} $th smallest element in the random sample is larger than all $ \smash{S_{(i)}} $. Intuitively, testing with the batch conformal $ p $-value rejects the null $ H_{k} $ if the quantile of the comparison scores $ \smash{S_{(\eta_{k})}^{(k)}} $ (which can be viewed as the test statistic for $ H_{k} $) is unusually large relative to the referencescores.

For example, if one chooses to use the median as the test statistic, one can set, e.g., $ \eta_{k}=\left\lceil 0.5\cdot n_{k}\right\rceil $. We remark that statistical inference for quantiles has been widely studied (Kosorok, 1999; Sgouropoulos et al., 2015; Yang & Zhao, 2017; Lou & Wu, 2025). However, we are not aware that our specific method has been widely studied. Specifically, as we will show, our method is valid in finite samples without distributional assumptions, while being powerful when the quantiles of the distributions differ. Thus, it has different properties from other methods such as those in Kosorok (1999) which are valid only asymptotically and require certain distributional assumptions.

When the comparison dataset has one data point, i.e., $ n_{k}=\eta_{k}=1 $, the batch conformal $ p $-value coincides with the standard conformal $ p $-value, given by $ \smash{(\sum_{i=1}^{n}}$$ \smash{{\mathbb{1}}\{{S_{1}^{(k)}\leqslant S_{i}}\}+1)}/{(n+1)}} $. Thus, the batch conformal $ p $-value can be viewed as an extension of the conformal $ p $-value. While the conformal $ p $-value can be used for outlier detection (Bates et al., 2023), the batch conformal $ p $-value can be used to detect ‘outlier groups’, as we will discuss in this work. We discuss the choice of the score $ s $ later. We further discuss the use of batch conformal $ p $-values for the special case of two-sample testing in § 5 of the Supplementary Material.

The idea of batch conformal $ p $-values is motivated by the predictive inference method for a quantile of multiple test points developed in Lee et al. (2025), leveraging classical results on the distribution of quantiles in finite populations; see, e.g., Wilks (1962). It follows from existing results that the formulas in (2) lead to marginally valid $ p $-values when the scores $ \smash{S_{1},\ldots,S_{n},S_{1}^{(k)},\ldots,S_{n_{k}}^{(k)}} $ are exchangeable. For completeness, we provide a self-contained proof of validity in the Supplementary Material. As a consequence, the $ p $-values $ p_{1},p_{2},\ldots,p_{K} $ form a vector of marginally valid $ p $-values.

Next, let $ I_{0}=\{k\in[K]\colon\text{} H_{k} \text{ is true}\} $ denote the set of group indices for which the null hypotheses from (1) hold, and let $ K_{0}=|I_{0}| $ denote the number of true nulls. We now prove our main result: that applying the Benjamini–Hochberg procedure with the batch conformal $ p $-values ensures valid false discovery rate control.

Theorem 1
(Main result: false discovery rate control). *Let the reference scores $ (S_{i})_{i\in[n]} $ and the comparison scores $ \smash{(S_{j}^{(k)})_{j\in[n_{k}],\,k\in[K]}} $ be almost surely all distinct, and suppose that the comparison score groups $ \smash{\{(S_{j}^{(k)})_{j\in[n_{k}]}\colon k\in[K]\}} $ are independent. Then, the batch conformal $ p $-values $ p_{1},p_{2},\ldots,p_{K} $ defined in (2) satisfy the positive regression dependence condition (Definition 1) on the set $ I_{0} $ of true nulls. Consequently, the Benjamini–Hochberg procedure at level $ \alpha $ (§ 2.1 and Algorithm {1} below), applied to $ (p_{k})_{k\in[K]} $, controls the false discovery rate at level $ K_{0}\alpha/K\leqslant\alpha $*.

Remark 2. The validity of batch conformal $ p $-values implies that we can, in fact, test the following weaker null hypotheses:

\begin{align*} H_{k}\colon\text{the } n+n_{k} \text{ scores }(S_{i})_{i\in[n]}\text{ and }(S_{j}^{(k )})_{j\in[n_{k}]}\text{ are exchangeable},\qquad k\in[K]. \end{align*}

The proof of Theorem 1 ensures that the Benjamini–Hochberg procedure applied to the batch conformal $ p $-values controls the false discovery rate for these nulls, if the comparison score groups $ \smash{\{(S_{j}^{(k)})_{j\in[n_{k}]}\colon k\in[K]\}} $ are independent conditional on the reference score group.

A statement $ H_{k}^{0} $, which asserts that the data points $ \smash{(X_{i},Y_{i})_{i\in[n]}} $ and $ \smash{(X_{j}^{(k)},Y_{j}^{(k)})_{j\in[n_{k}]}} $ are exchangeable, implies the above $ H_{k} $, provided that the score function $ s $ is either fixed or constructed using a data split. Therefore, the proposed procedure also provides FDR control for the nulls $ H_{k}^{0} $, $ k\in[K] $.

Remark 3. The positive regression dependence property established in Theorem 1 also has implications for the global null testing problem. Specifically, consider the problem of testing whether there exists at least one comparison dataset whose distribution differs from that of the reference data. A straightforward approach to control the Type I error is the Bonferroni test, which accommodates arbitrary dependence among $ p $-values and can therefore be applied with any existing two-sample $ p $-values. However, the Bonferroni test is well known to be conservative. In contrast, the Simes test offers a less conservative alternative, but requires certain conditions on the $ p $-values. Sarkar (2008) showed that the Simes test remains valid under positive regression dependence, implying that, by Theorem 1, the batch conformal $ p $-values can also be used within the Simes test while ensuring valid Type I error control.

*Algorithm* 1. Distribution-shift detection with batch conformal $ p $-values.   **Input:** Reference data $ \mathcal{D}_{n}=\{X_{1},X_{2},\ldots,X_{n}\} $. Comparison sets $ \smash{(X_{1}^{(k)},X_{2}^{(k)},}\ldots, $
$ \smash{X_{n_{k}}^{(k)})_{1\leqslant k\leqslant K}} $ . Score function $ s\colon\mathcal{X}\rightarrow\mathbb{R} $. Target level $ \alpha $. Rank vector $ (\eta_{1},\ldots,\eta_{K})\in[n_{1}]\times $

$ \cdots\times[n_{K}] $ .

**Step 1:** Compute the scores $ S_{i}=s(X_{i}) $ for $ 1\leqslant i\leqslant n $ and $ \smash{S_{i}^{(k)}=s(X_{i}^{(k)})} $ for $ 1\leqslant k\leqslant $

$ K $ and $ 1\leqslant i\leqslant n_{k} $.

**Step 2:** Compute the batch conformal $ p $-values $ p_{1},\ldots,p_{K} $ as

\begin{align*} p_{k} &=\sum\limits_{i=1}^{n}\left\{\binom{i+\eta_{k}-2}{\eta_{k}-1}\binom{n +n_{k}-i-\eta_{k}+1}{n_{k}-\eta_{k}}\bigg{/}{\binom{n+n_{k}}{n_{k}}}\right\} \cdot{\mathbb{1}}\{{S_{(\eta_{k})}^{(k)}\leqslant S_{(i)}}\} \\ &\quad+{\binom{n+\eta_{k}-1}{\eta_{k}-1}}\bigg{/}{\binom{n+n_{k}}{ n_{k}}},\qquad k\in[K], \end{align*}

and sort them as $ p_{(1)}\leqslant\cdots\leqslant p_{(K)} $.

**Step 3:** Run the Benjamini–Hochberg procedure to find $ k^{*}=\max\{k\colon p_{(k)}\leqslant{k}\alpha/{K}\}. $

**Return:** Rejection set $ \mathcal{R}=\{k\in[K]\colon p_{k}\leqslant p_{(k^{*})}\} $ ($ \mathcal{R}=\emptyset $ if $ \min_{1\leqslant k\leqslant K}{K}p_{(k)}/{k}\gt\alpha $).

The overall procedure is described in Algorithm 1Algorithm 1 and the proof of Theorem 1 is deferred to Supplementary Material. It introduces a novel technique that differs from proof techniques used in related results, such as those in Bates et al. (2023). Our approach introduces a technique for constructing random variables $ \smash{(\tilde{R}_{1},\ldots,\tilde{R}_{m})} $ and $ \smash{(\hat{R}_{1},\ldots,\hat{R}_{m})} $ inductively over $ m $, ensuring that they have the same distribution as the ranks of the test scores conditional on two different values of $ p_{k} $, while also ensuring that $ \smash{(\tilde{R}_{1},\ldots,\tilde{R}_{m})\preceq(\hat{R}_{1},\ldots,\hat{R}_ {m})} $ holds almost surely. To enable this construction, we show that the conditional distribution of the ranks $ R_{t-1} $, given $ R_{t} $ and the set of scores, is stochastically increasing in $ R_{t} $ (Lemma 2 in the Supplementary Material). Strassen’s theorem (Strassen, 1965) is then applied repeatedly to construct appropriate random variables $ \smash{\tilde{R}_{t-1}\leqslant\hat{R}_{t-1}} $ having the same conditional distributions as $ R_{t-1} $, given the set of scores and two appropriate values of $ R_{t} $. This construction may have broader applications beyond the scope of our paper.

### 2.4. Choice of the score function

By Theorem 1, Algorithm 1Algorithm 1 ensures valid false discovery rate control for any score function constructed independently of the reference and comparison data. However, the power of the test depends on this choice. Here, we discuss several possible choices for the score function across different scenarios.

1. *One-dimensional outcome*. Let the observed outcomes be scalars: $ Y_{1},\cdots,Y_{n}\in\mathbb{R} $ for the reference set, and $ \smash{Y_{1}^{(k)},\ldots,Y_{n_{k}}^{(k)}\in\mathbb{R},\,k\in[K]} $ for each comparison group $ k\in[K] $. To detect groups with a distribution shift, one can set the score function simply as $ s(y)=y $ or $ s(y)=-y $ for all $ y $, depending on the direction of the shift of interest. For two-sided detection, one direct option is to split the reference data into two sets, use one set to construct an estimate $ \hat{\mu} $ of the ‘centre’ (e.g., mean or median) and then set the score function as $ s\colon y\mapsto|y-\hat{\mu}| $ to run the procedure on the second split.

2. *One-dimensional outcome with side information*. For the reference group, $ (X_{1},Y_{1}),\ldots,(X_{n},Y_{n})\in\mathbb{R}^{d}\times\mathbb{R} $, and similarly for the comparison groups, where $ X_{1},\ldots,X_{n} $ represent side information whose distribution is identical across groups. In this setting, standard nonconformity scores (Vovk et al., 2005) can be used; for example, one can apply data splitting to the reference set and use one split to construct scores such as (Romano et al., 2019)


\begin{align*} s\colon(x,y)\mapsto|y-\hat{\mu}(x)|\quad\mathrm{or}\quad s\colon(x,y)\mapsto\max \{\hat{q}_{\alpha/2}(x)-y,y-\hat{q}_{1-\alpha/2}(x)\}, \end{align*}

where $ \hat{\mu}(\cdot) $ is an estimator of the conditional mean $ \mathbb{E}[Y\mid X] $, and $ \hat{q}_{\alpha/2}(\cdot) $ and $ \hat{q}_{1-\alpha/2}(\cdot) $ are estimators of the $ \alpha/2 $ and $ 1-\alpha/2 $ conditional quantiles. Being error measures for an estimator fitted to be small under the null (reference) distribution, these scores are expected to be larger when there is a shift.

3. *Multivariate outcome variable*. Now consider features with multivariate outcomes: $ (X_{1},Y_{1}),\ldots,(X_{n},Y_{n})\in\mathbb{R}^{d}\times\mathbb{R}^{p} $, where each $ Y_{i}=(Y_{i1},\ldots,Y_{ip})^{\mathrm{\scriptscriptstyle T}}\in\mathbb{R}^{p} $, and similarly for the comparison groups. Here, $ X_{1},\ldots,X_{n} $ denote the side information whose distribution does not differ across groups. One option is to use the training data (one split of the reference dataset) to construct $ \hat{\mu}_{1},\dots,\hat{\mu}_{p} $ such that $ \hat{\mu}_{j}(X) $ predicts the $ j $th component of the outcome $ Y $, and then combine them. For example, one can set the score $ s\colon\mathbb{R}^{d}\times\mathbb{R}^{p}\rightarrow\mathbb{R} $ as $ \smash{s\colon(x,y)\mapsto\sum_{j=1}^{p}|y_{j}-\hat{\mu}_{j}(x)|^{2}=\|\hat{r} (x,y)\|^{2},} $ where $ \smash{\hat{r}(x,y)=\{|y_{j}-\hat{\mu}_{j}(x)|\}_{1\leqslant j\leqslant p}} $ denotes the residual vector.

To address potentially different scales and dependencies between the components, one can alternatively set the score as $ s\colon(x,y)\mapsto\hat{r}(x,y)^{\mathrm{\scriptscriptstyle T}}\hat{S}^{-1} \cdot\hat{r}(x,y), $ where $ \hat{S} $ denotes the sample covariance matrix of the residual vectors evaluated on training data. To better capture the dependence structure within the outcome vector, one can also consider constructing the residual vector in the form


\begin{align*} &\hat{r}\colon(x,y)\mapsto\{y_{1}-\hat{\mu}_{1}(x),y_{2}-\hat{\mu}_{2}(x,y_{1}),y_{3}-\hat{\mu}_{3}(x,y_{1},y_{2}),\dots,y_{p}\nonumber\\ &\quad -\hat{\mu}_{p}(x,y_{1},\dots,y_ {p-1})\}^{\mathrm{\scriptscriptstyle T}}, \end{align*}

with $ \hat{\mu}_{j}\colon\mathbb{R}^{d}\times\mathbb{R}^{j-1}\rightarrow\mathbb{R} $ constructed accordingly for each $ j\in[p] $.

### 2.5. Choice of the hyperparameters $ \eta_{k} $

The proposed procedure involves hyperparameters $ (\eta_{k})_{k\in[K]} $, which must be selected by the practitioner. A natural default choice is the median, i.e., setting $ \eta_{k}=\left\lceil n_{k}/2\right\rceil $, although other choices may be appropriate depending on the target of interest. For example, if $ X $ is one dimensional and $ s(x)=x $, and the goal is to detect shifts in the tails of the outcome distribution, a larger value such as $ \eta_{k}=\left\lceil 0.9\cdot n_{k}\right\rceil $ can be used.

Alternatively, for more complex settings, e.g., with multivariate $ X $ and nonidentity scores, one may consider tuning the hyperparameter based on the data to increase power. For example, for each $ k\in[K] $, let $ \smash{G_{k}^{0}=(X_{1}^{(k)},\ldots,X_{m_{k}}^{(k)})} $ denote a split of the comparison data $ G_{k} $, with $ m_{k}< n_{k} $, and let $ \smash{G^{0}=(X_{1},\ldots,X_{m})} $ be a split of the reference data (which may be the same split used for constructing the score function). Then, for values $ q $ in a grid, e.g., $ (0.1,0.2,\ldots,0.9) $, compute the (one-sided) difference between the $ q $-sample quantiles of the score sets $ \smash{\{s(X_{1}^{(k)}),\ldots,s(X_{m_{k}}^{(k)})\}} $ and $ \smash{\{s(X_{1}),\ldots,s(X_{m})\}} $, and select the value $ q_{k} $ that maximizes this difference. The remaining split $ \smash{G_{k}^{1}=(X_{m_{k}+1}^{(k)},\ldots,X_{n_{k}}^{(k)})} $ is then used for inference, with $ \eta_{k}=\left\lceil q_{k}\cdot(n_{k}-m_{k})\right\rceil $.

Remark 4. In settings where the variable $ X $ represents more than side information and its distribution differs across groups, the method described above may not be reliable, as the procedure could detect a shift in the marginal distribution of $ X $ rather than that of $ Y $. In such cases, the use of $ X $ in the inference should be avoided; instead, one can construct scores based solely on the target variable $ Y $.

Remark 5. In § 4 of the Supplementary Material, we provide a power analysis in a simple score-level model, which offers an intuitive illustration of the types of shifts that the proposed procedure is well suited to detect.

## 3. Simulations

### 3.1. Simple case: detecting shifts in a one-dimensional outcome

**Figure 2:**
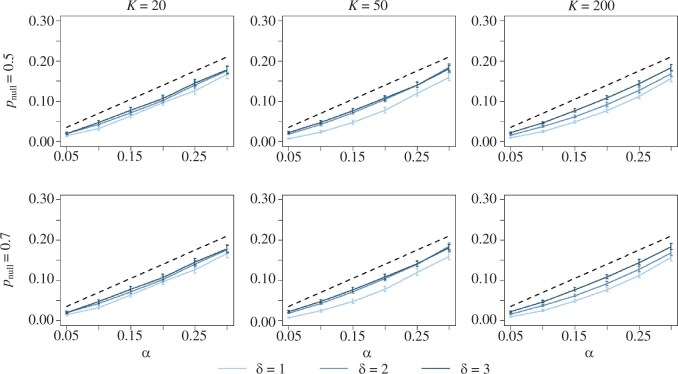
False discovery rate of Algorithm 1 across various signal strengths and numbers of comparison groups, for true-null proportions $ p_{\mathrm{null}}=0.5 $ and $ p_{\mathrm{null}}=0.7 $. The dotted line corresponds to the theoretical bound $ p_{\mathrm{null}}\cdot\alpha $from Theorem 1.

We first illustrate the performance of Algorithm 1Algorithm 1 in a simple setting with a one-dimensional outcome and no covariates. We generate the reference dataset of size $ n=100 $ from the $ \mathcal{N}(0,3^{2}) $ distribution. Next, we generate multiple comparison datasets, with sizes $ (n_{k})_{1\leqslant k\leqslant K} $ drawn uniformly in advance between 30 and 50. The simulation is run under two null proportions, 0.5 and 0.7, and three numbers of comparison groups: 20, 50 and 200. For the nonnull groups, the data are drawn from $ \mathcal{N}(\delta,3^{2}) $, using signal strengths $ \delta=1,2,3 $. We use the median as the test statistic for each group, i.e., $ \eta_{k}=\left\lceil n_{k}/2\right\rceil $. The results are shown in Fig. 2, illustrating that the proposed procedure tightly controls the false discovery rate at the theoretically proven level, supporting the conclusion of Theorem 1.

Next, we compare the performance of Algorithm 1Algorithm 1 with an ‘oracle procedure’ that applies the Benjamini–Hochberg procedure to $ p $-values obtained from two-sample $ z $-tests, given by


**Figure 3:**
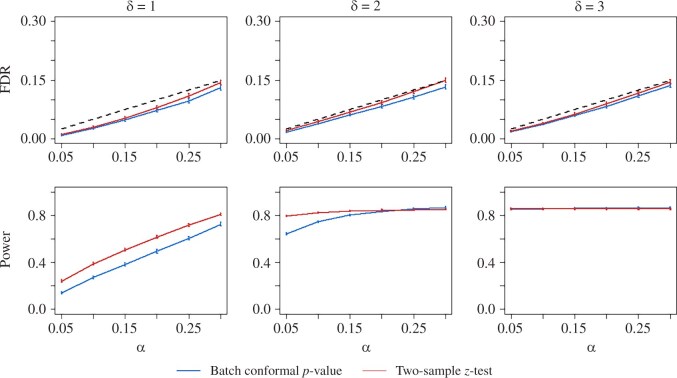
False discovery rate and power of Algorithm 1 and of the procedure using $ p $-values from the two-sample $ z $-test, across various signal strengths, for a true-null proportion of 0.5. The dotted line corresponds to the theoretical bound $ 0.5\alpha $ from Theorem 1.

\begin{align*} p_{k}=\Phi\bigg[(\bar{X}_{\mathrm{ref}}-\bar{X}_{k})\bigg{/}\bigg\{\sigma{ {\bigg(\frac{1}{n}+\frac{1}{n_{k}}}\bigg)^{1/2}}\bigg\}\bigg],\qquad k =1,2,\ldots,K, \end{align*}

where $ \bar{X}_{\mathrm{ref}} $ and $ \bar{X}_{k} $ represent the sample means of the reference dataset and the $ k $th comparison dataset, respectively, and $ \Phi $ denotes the cumulative distribution function of a standard normal random variable. Here, the variance is assumed to be known, with $ \sigma=3 $ in our examples. These $ p $-values satisfy positive regression dependence (see the Supplementary Material), and thus the Benjamini–Hochberg procedure guarantees false discovery rate control. We generate the data as before under $ \delta=1,2,3 $ and $ K=50 $. The results are shown in Fig. 3, illustrating that our proposed procedure attains power comparable to that of the oracle procedure.

**Figure 4:**
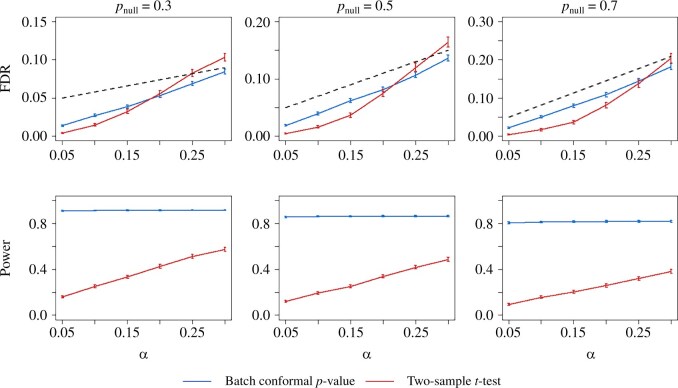
False discovery rate and power of Algorithm 1 and of the procedure using $ p $-values from the two-sample $ t $-test, under a nonnormal null distribution, for true-null proportions of $ 0.3,0.5 $ and 0.7. The dotted line corresponds to thetheoretical bound from Theorem 1.

We also provide results for the case where the true distribution is nonnormal, in which approaches based on the normal model assumption may not perform well. We repeat the same simulation with the following null and alternative distributions:


\begin{align*} &\quad\quad\quad X\sim 0.5\cdot{\rm Cauchy}(0,1)+0.5\cdot{\rm Un}[-1,1]\quad\text {(null)}, \\ &X\sim\delta+0.5\cdot{\rm Cauchy}(0,1)+0.5\cdot{\rm Un}[-1,1] \quad\mathrm{(alternative)}, \end{align*}

where we set $ \delta=1 $. We compare the results of Algorithm 1Algorithm 1 with those obtained using two-sample $ t $-test $ p $-values. Figure 4 summarizes the results for true-null proportions of 0.3, 0.5 and 0.7. The false discovery rate of the procedure based on $ z $-test $ p $-values occasionally exceeds the bound $ (\text{true-null proportion})\cdot\alpha $, but remains controlled at level $ \alpha $. However, its power is significantly lower than that of the distribution-free Algorithm 1Algorithm 1.

**Figure 5:**
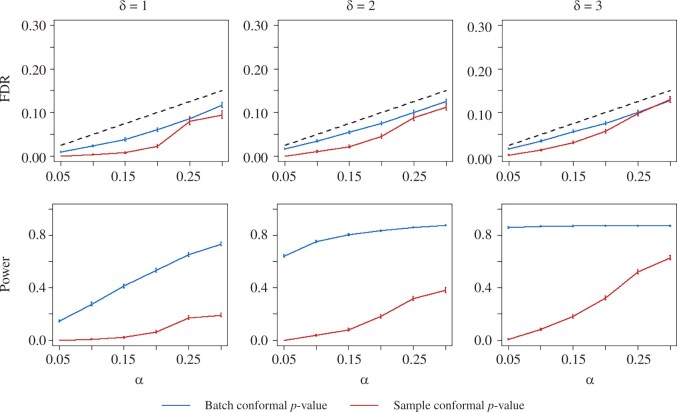
False discovery rate and power of the procedure using batch conformal $ p $-values and of the procedure using subsampling conformal $ p $-values, across different signal strengths. The dotted line corresponds to the theoretical boundfrom Theorem 1.

Next, we compare the performance of the proposed procedure with the method discussed in § 2.2, which uses the conformal $ p $-value constructed by subsampling. Figure 5 illustrates that both methods control the false discovery rate at the desired level, but thesubsampling-based approach often leads to low power. Intuitively, this is likely because using only a single sample in the nonnull case does not provide sufficient evidence to conclude that the sampling distribution differs from that of the reference set.

### 3.2. Detecting shifts in a multivariate outcome

Next, we perform experiments in a setting aimed at detecting shifts in a multivariate outcome. We generate a training set $ (X_{i}^{\mathrm{train}},Y_{i}^{\mathrm{train}})_{1\leqslant i\leqslant 100}\subset \mathbb{R}^{10}\times\mathbb{R}^{5} $ and a calibration dataset $ (X_{i},Y_{i})_{1\leqslant i\leqslant 50}\subset\mathbb{R}^{10}\times\mathbb{R} ^{5} $, of sizes 100 and 50, respectively, from the following distribution:


\begin{gather*} X\sim{\rm Un}([0,1]^{10}), \\ (Y_{1},Y_{2},Y_{3})\mid X\sim\mathcal{N}(\beta_{1}^{\mathrm{ \scriptscriptstyle T}}X,\Sigma), \\ Y_{4}\mid X,Y_{1},Y_{2},Y_{3}\sim{\rm Ga}\{(Y_{1}^{2}+Y_{2}^{2}) /2,2\}, \\ Y_{5}\mid X,Y_{1},Y_{2},Y_{3},Y_{4}\sim{\rm Be}\big(|Y_{1}|,|Y _{2}|+\tfrac{1}{2}|Y_{3}|\big). \end{gather*}

Here, each entry of $ \beta_{1}\in\mathbb{R}^{10\times 3} $ is generated independently from a uniform distribution over $ [0,1] $ and fixed in advance, while $ \Sigma $ is a $ 3\times 3 $ matrix with entries $ \Sigma_{ij}=2-|i-j| $ for all $ i,j\in[3] $. Next, we generate 50 comparison groups, with group sizes sampled in advance from the distribution $ 5+{\rm Po}(20) $. The data points in each group are drawn from one of five distributions, parameterized by $ t\in\{0,1,2,3,4\} $ assigned to each group:


\begin{gather*} X\sim{\rm Un}([0,1]^{10}), \\ (Y_{1},Y_{2},Y_{3})\mid X\sim\mathcal{N}(\beta_{1}^{\mathrm{ \scriptscriptstyle T}}X+t\cdot|\beta_{2}^{\mathrm{\scriptscriptstyle T}}X|^{2},\Sigma), \\ Y_{4}\mid X,Y_{1},Y_{2},Y_{3}\sim{\rm Ga}\{(t\cdot|\beta_{3}^{ \mathrm{\scriptscriptstyle T}}X|+Y_{1}^{2}+Y_{2}^{2})/2,2\}, \\ Y_{5}\mid X,Y_{1},Y_{2},Y_{3},Y_{4}\sim{\rm Be}\big(|Y_{1}|,|Y _{2}|+\tfrac{1}{2}|Y_{3}|\big). \end{gather*}

Here $ t=0 $ corresponds to the null distribution. The true-null proportion is set to 0.5; that is, 25 groups are drawn from the distribution with $ t=0 $, while $ t=1,2,3,4 $ are assigned to ten, five, five and five groups, respectively. We construct estimators $ (\hat{\mu}_{j})_{j\in[5]} $ of the conditional means of $ (Y_{j})_{j\in[5]} $ by fitting the training data with random forest regression. Let $ \hat{S} $ be the sample covariance matrix of the resulting residual vectors $ \hat{r} $ evaluated on the training set. We run Algorithm 1Algorithm 1 with three choices of score, with $ \eta_{k}=\left\lceil n_{k}/2\right\rceil $ for all groups.

*Score* A: $ s\colon(x,y)\mapsto\hat{r}(x,y)^{\mathrm{\scriptscriptstyle T}}\hat{S}^{-1} \hat{r}(x,y) $, where


\begin{align*} \hat{r}\colon(x,y)\mapsto\{y_{1}-\hat{\mu}_{1}(x),y_{2}-\hat{\mu}_{2}(x),y_{3} -\hat{\mu}_{3}(x),y_{4}-\hat{\mu}_{4}(x),y_{5}-\hat{\mu}_{5}(x)\}. \end{align*}

*Score* B: $ s\colon(x,y)\mapsto\hat{r}(x,y)^{\mathrm{\scriptscriptstyle T}}\hat{S}^{-1} \hat{r}(x,y) $, where


\begin{align*} \hat{r}\colon(x,y)\mapsto\{ & y_{1}-\hat{\mu}_{1}(x),y_{2}-\hat{\mu}_{2}(x,y_{1}),y_{3}-\hat{ \mu}_{3}(x,y_{1},y_{2}),y_{4}-\hat{\mu}_{4}(x,y_{1},y_{2},y_{3}), \\ & y_{5}-\hat{\mu}_{5}(x,y_{1},y_{2},y_{3},y_{4})\}. \end{align*}

*Score* C:$ s\colon(x,y)\mapsto\hat{r}(x,y)^{\mathrm{\scriptscriptstyle T}}\hat{S}^{-1} \hat{r}(x,y) $, where


\begin{align*} \hat{r}\colon(x,y)\mapsto\{ & y_{5}-\hat{\mu}_{1}(x),y_{4}-\hat{\mu}_{2}(x,y_{5}),y_{3}-\hat{ \mu}_{3}(x,y_{4},y_{5}),y_{2}-\hat{\mu}_{4}(x,y_{3},y_{4},y_{5}), \\ & y_{1}-\hat{\mu}_{5}(x,y_{2},y_{3},y_{4},y_{5})\}. \end{align*}

Intuitively, score A does not capture the dependence structure between the components of the outcome, while scores B and C do. However, score C uses a misspecified regression model for the outcomes.

**Figure 6:**
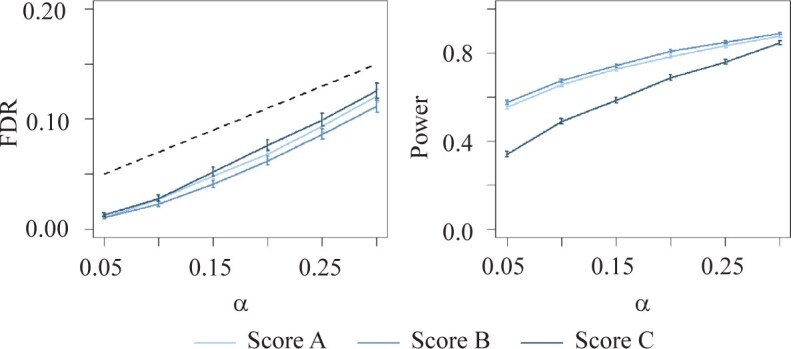
False discovery rate and power of Algorithm 1 for different scores. The dotted line corresponds to the theoretical bound $ 0.5\alpha $ from Theorem 1.

We repeat the process of generating the reference and comparison datasets and running the procedure 500 times. The results are summarized in Fig. 6, illustrating that the proposed procedure controls the false discovery rate at the level provided by Theorem 1, and that the power of the tests is similar for different choices of score.

**Figure 7:**
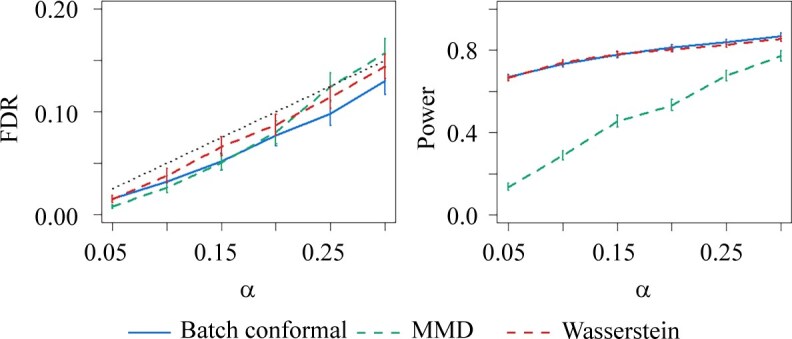
False discovery rate and power of Algorithm 1 and of methods using the MMD and Wasserstein distance statistics. The dotted line corresponds to the theoretical bound $ 0.5\alpha $.

Next, we provide comparisons with methods without theoretical guarantees, namely the Benjamini–Hochberg procedure combined with two popular methods for two-sample testing: the kernel two-sample test with maximum mean discrepancy (MMD) statistic (Gretton et al., 2012) and the Wasserstein-distance-based method (Ramdas et al., 2017). We follow the same setting as above, with score A. The results are shown in Fig. 7.

The results show that the proposed procedure outperforms the MMD-based method and achieves performance comparable to the Wasserstein-distance-based method. These findings suggest that attaining strict distribution-free, finite-sample control of the false discovery rate does not come at the expense of empirical performance, i.e., high power can be achieved in a theoretically reliable and computationally simple manner.

### 3.3. Two-sample test for distributional equality

Here, we provide simple simulation results for batch conformal $ p $-value-based testing in the two-sample setting, and compare with other distribution-free two-sample tests, namely the permutation test and the rank-sum test. See § 5 of the Supplementary Material for further discussion of this setting.

We generate reference samples of size $ n=30 $ from $ \mathcal{N}(0,1) $ and comparison samples of size $ m=30 $ from $ \mathcal{N}(0,3) $. The two distributions thus differ in scale, but not in location, i.e., they have the same mean and median. We then construct three $ p $-values:

1. the batch conformal $ p $-value, computed according to (5) in the Supplementary Material, with $ \eta=\left\lceil q\cdot m\right\rceil $;
2. the permutation test $ p $-value, computed according to (2) in the Supplementary Material, with test statistic $ T(X_{1:n+m})=Q_{q}(X_{1:n})-Q_{q}(X_{(n+1):(n+m)}) $ and $ L=1000 $ permutations;
3. the rank-sum test $ p $-value, computed with wilcox.test function.

Here, $ Q_{q} $ denotes the $ q $-quantile function. We repeat these steps 500 times and compute the power of the three methods for two choices of $ q $: $ q=0.8 $ and $ q=0.5 $. The former corresponds to comparing the 0.8 quantiles of the two samples, which makes the batch conformal test and the permutation test more likely to detect the difference. The latter represents a poor choice, since the resulting test statistic (the median) is the same for both distributions, making it harder to distinguish between them. The rank-sum test does not involve such a hyperparameter, and therefore remains the same in both comparisons.

**Figure 8:**
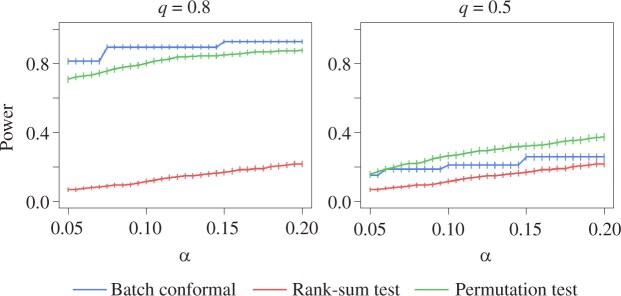
Power of the batch conformal $ p $-value-based test, the rank-sum test and the permutation test, with two choicesof test statistic, along with error bars.

The results are shown in Fig. 8. When a tail quantile ($ q=0.8 $) is used as the test statistic, both the batch conformal $ p $-value-based test and the permutation test show significantly higher power than the rank-sum test, as expected, since the rank-sum test does not capture differences in the tails. Notably, the batch conformal $ p $-value test even achieves higher power than the permutation test. When the median ($ q=0.5 $) is used, both methods have low power, but still achieve power higher than the rank-sum test. In summary, the test based on the batch conformal $ p $-value has a significant advantage over the rank-sum test when the difference between distributions lies mainly in the tails, and attains power comparable to the permutation test, while being computationally muchcheaper.

## 4. Empirical illustration

### 4.1. Overview

We apply the proposed procedure to the HALT-C dataset (Snow, 2024), which contains data from patients with chronic hepatitis C who were randomly assigned to either receive peginterferon treatment or not. (Experiments for the CPS data are provided in the Supplementary Material.) The data were collected from ten study sites. We consider the following two tasks:

(i) comparison of treatment effects across different patient groups;
(ii) comparison of patient-level baseline characteristics across ten study sites.

In the first task, rather than comparing distributions using observed data points, we consider the more challenging task of comparing within-group treatment effects without direct access to the data points, as counterfactuals are unavailable.

### 4.2. Identifying patient groups with larger treatment effects

We first investigate the effect of the treatment on platelet counts measured after nine months. After removing data points lacking treatment or platelet information, the sample size is 844.

We partition the sample based on age, BMI and sex. Age is divided into four groups: $ < 40 $, 40–49, 50–59 and $ \geqslant 60 $. Similarly, BMI is categorized into four groups: $ < 25 $, 25–29.9, 30–34.9 and $ > 35 $. As a result, the total number of groups formed by these three categorical variables is $ 4\cdot 4\cdot 2=32 $. Among these groups, we exclude any group with a sample size that is too small; specifically, those for which either the control arm or the treated arm has size less than or equal to one. Among the remaining 26 groups, we select the group age 40–49 / BMI 25–29.9 / male, which has the largest sample size, as the reference group. The other $ K=25 $ groups are used as comparison groups.

Throughout this section, we denote the counterfactual distributions of the comparison groups by $ P_{k,0} $ and $ P_{k,1} $ for $ k\in[K] $, and those of the reference group by $ P_{0,0} $ and $ P_{0,1} $. Furthermore, we denote the cumulative distribution function of $ P_{k,0} $ by $ F_{k,0} $ for each $ k\in\{0\}\cup[K] $. Let $ Y_{k1},\dots,Y_{kn_{k}} $ represent the outcomes of the treated individuals in the $ k $th group, and let $ Y_{1},\dots,Y_{n} $ denote the outcomes in the reference group.

Assuming for now that the cumulative distribution functions are known, construct the quantities $ \smash{S_{i}^{(k)}=F_{k,0}(Y_{ki})} $ for $ i\in[n_{k}] $ in the comparison groups, and $ S_{i}=F_{0,0}(Y_{i}) $ for $ i\in[n] $ in the reference group. We use the identity score $ s(y)=y $. The detection procedure based on these cumulative-distribution-function-based quantities tests


(3) \begin{align*} H_{k}\colon F_{k,0}(Y_{k})\stackrel{{\scriptstyle{\rm\small D} }}{{=}}F_{0,0}(Y), \quad\mathrm{where}\quad Y_{k}\sim P_{k,1}\text{ and }Y\sim P_{0,1},\text{ for } k\in[K]. \end{align*}

The test based on the batch conformal $ p $-value rejects the null when an appropriate quantile of the comparison scores $ (F_{k,0}(Y_{k,i}))_{i\in[n_{k}]} $ is larger than the corresponding quantile of the reference scores $ \smash{\{F_{0,0}(Y_{i})\}_{i\in[n]}} $. When the potential outcomes follow continuous distributions, the null hypothesis $ H_{k} $ in (3) is equivalent to $ \smash{F_{k,0}\circ({F_{k,1}}^{-1})=F_{0,0}\circ({F_{0,1}}^{-1})} $, where $ \cdot^{-1} $ denotes the inverse function. Here, $ F_{k,0}\circ({F_{k,1}}^{-1}) $ is the treated-to-control quantile-quantile map in the $ k $th group, also known as the (inverse) response map (Doksum, 1974; Doksum & Sievers, 1976). This map has been used to quantify treatment effects in, e.g., Athey et al. (2023).

In practice, the cumulative distribution functions must be estimated using data from the control arm, for example by the empirical cumulative distribution function. (It is also possible to use an external database with a much larger sample size to construct more accurate estimates of the cumulative distribution functions, since only the control-group cumulative distribution function is required; for example, the distribution of standard platelet counts in untreated individuals in our example.) For each $ k\in\{0\}\cup[K] $, denote by $ \smash{Y_{k1}^{(0)},\dots,Y_{kn_{k}^{(0)}}^{(0)}} $ the outcomes of the control individuals in the $ k $th group, and let $ Y^{(0)}_{1},\dots,Y^{(0)}_{n^{(0)}} $ denote the outcomes in the control arm of the reference group. For each $ k\in\{0\}\cup[K] $, let the empirical cumulative distribution function of the outcomes in the control arm of group $ k $ be $ \hat{F}_{k,0} $. We then consider the following null hypotheses conditional on the control arms, so that the empirical cumulative distribution functions can be treated as fixed:


(4) \begin{align*} \tilde{H}_{k}\colon\hat{F}_{k,0}(Y_{k})\stackrel{{\scriptstyle {\rm\small D} }}{{=}}\hat{F}_{0,0}(Y)\,\mid Y_{k1}^{(0)},\dots,Y_{kn_{k}^{(0)}}^{( 0)},\,Y^{(0)}_{1},\dots,Y^{(0)}_{n^{(0)}}, \end{align*}

where $ Y_{k}\sim P_{k,1} $ and $ Y\sim P_{0,1} $, for $ k\in[K] $. Our procedure provides exact false discovery rate control for testing the hypotheses (4). While the hypotheses in (4) differ from those in (3), they nonetheless capture the same goal, detecting groups with larger treatment effects. The formulation in terms of hypotheses (4) can be viewed as the finite-population counterpart of the formulation based on (3).

Table 1 shows the results from the method using cumulative-distribution-function-based scores. As in the previous experiment, the two quartiles and the median are used as test statistics. The number of detected groups is larger for higher quantiles, especially for $ Q_{3} $, suggesting that there are larger differences in treatment effects between the comparison and reference groups at higher quantiles of the treated outcomes.

**Table 1:** *Results for the HALT-C dataset: selected groups at levels*  $ \alpha=0.05,0.1 $  *and* 0.2, *out of* 25 *groups based on age, BMI and sex.The reference group corresponds to age* 40–49/*BMI* 25–29.9/*male*

			$ Q_{1} $			$ Q_{2} $			$ Q_{3} $		
Age	BMI	Sex	0.05	0.1	0.2	0.05	0.1	0.2	0.05	0.1	0.2
$ < 40 $	$ \geqslant 35 $	M	–	–	–	–	–	✓	–	✓	✓
40–49	$ < 25 $	M	–	–	–	–	–	–	–	✓	✓
40–49	25–29.9	F	–	–	–	–	–	–	✓	✓	✓
40–49	30–34.9	M	–	✓	✓	–	–	–	–	–	–
40–49	30–34.9	F	–	–	✓	–	–	–	–	–	–
40–49	$ \geqslant 35 $	M	–	✓	✓	–	–	✓	–	–	✓
50–59	$ < 25 $	F	–	–	–	–	–	✓	✓	✓	✓
50–59	25–29.9	M	–	–	–	✓	✓	✓	✓	✓	✓
50–59	25–29.9	F	–	✓	✓	–	–	✓	–	–	✓
50–59	30–34.9	M	–	✓	✓	–	✓	✓	✓	✓	✓
50–59	30–34.9	F	–	–	✓	–	✓	✓	–	–	–
50–59	$ \geqslant 35 $	M	–	–	–	–	–	✓	–	✓	✓
$ \geqslant 60 $	30–34.9	M	✓	✓	✓	✓	✓	✓	✓	✓	✓
$ \geqslant 60 $	30–34.9	F	–	✓	✓	–	–	–	–	–	–
$ \geqslant 60 $	$ \geqslant 35 $	F	–	–	–	✓	✓	✓	✓	✓	✓

**Figure 9:**
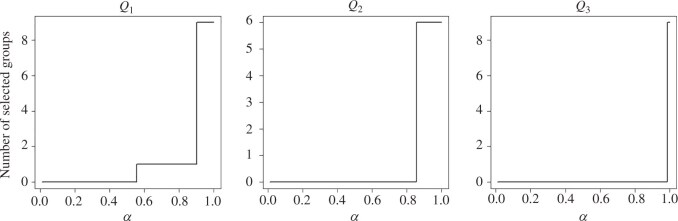
Results for the HALT-C dataset: the number of study sites selected by Algorithm 1, for different values ofthe level $ \alpha $.

### 4.3. Identifying study sites with different population characteristics

Next, we compare the population composition across the ten study sites in terms of patients’ baseline characteristics. We conduct the test based on age, BMI, sex and baseline platelet counts (distinct from the ‘platelet counts after treatment’ used in the previous experiment). The study site with the largest sample size (284) is chosen as the reference group, and the remaining nine sites are set as the comparison groups. In other words, denoting the joint distribution of age, BMI, sex and platelet counts for the $ k $th comparison site as $ P^{(k)} $ and for the reference site as $ P $, we test


\begin{align*} H_{k}\colon P^{(k)}=P,\qquad k=1,2,\ldots,9. \end{align*}

To construct the score function, we split the reference data into two sets, each of size 142, and use one split to compute the estimates $ \hat{\mu}=(\hat{\mu}_{\mathrm{age}},\hat{\mu}_{\mathrm{BMI}},\hat{\mu}_{\mathrm{sex} },\hat{\mu}_{\mathrm{pl}}) $, which are simply the sample means of age, BMI, sex (encoded as a binary variable) and platelet counts, respectively. We also compute the sample covariance matrix $ \hat{S} $ on the same split, and define the score function as


\begin{align*} s(x)=s\{(x_{\mathrm{age}},x_{\mathrm{BMI}},x_{\mathrm{sex}},x_{\mathrm{pl}})\}=(x-\hat {\mu})^{\mathrm{\scriptscriptstyle T}}\hat{S}^{-1}(x-\hat{\mu}). \end{align*}

The second split is used to compute the $ p $-values along with the comparison datasets.

Figure 9 presents the number of rejections from Algorithm 1Algorithm 1 at levels $ \alpha=0.01,0.02,\dots,1 $, with three choices of test statistic: the lower and upper quantiles, and the median. Specifically, for each group, we construct the $ p $-value using $ \eta_{k}=\left\lceil 0.25\cdot n_{k}\right\rceil $, $ \left\lceil 0.5\cdot n_{k}\right\rceil $ or $ \left\lfloor 0.75\cdot n_{k}\right\rfloor $ in (2). None of the comparison sites is found to have a different distribution from the reference site unless the target false discovery rate control level $ \alpha $ is set to a large value, which is not typically used in multiple-testing procedures. Thus, nosignificant differences are detected in the population composition across the ten sites, providing one component of justification for using a site-pooled sample in various analyses.

## 5. Discussion

Several open questions remain regarding the application of batch conformal $ p $-values. For instance, it can be of interest to test for pairs of groups, beyond comparing every dataset with a single reference dataset (see, e.g., Dunnett, 1955). Is there a way to re-use the data in each group to construct $ p $-values, and can we construct a multiple-testing procedure that outputs a consistent decision, i.e., such that the individual decisions do not contradict each other? These questions are left for future exploration.

## Supplementary Material

asag025_Supplementary_Data

## Supplementary material

The Supplementary Material includes additional experimental results and proofs of the theorems. The code to reproduce the experiments is available at https://github.com/yhoon31/batch\_conformal.
