# Supplementary material for: Finding distributions that differ, with false discovery rate control
Source: Biometrika. 2026 Apr 4;113(2):asag025. doi: 10.1093/biomet/asag025 (PMC13245922; doi:10.1093/biomet/asag025)
Supplement: asag025_Supplementary_Data [file asag025_supplementary_data.pdf]

Biometrika (2025), **112**, 1, *p*. 1
Printed in Great Britain Advance Access publication on 14 February 2025

Supplementary Material for "Finding Distributions that Differ, with False Discovery Rate Control"

BY YONGHOON LEE
Department of Statistics and Data Science, the Wharton School, University of Pennsylvania, Philadelphia, PA, USA
yhoony31@wharton.upenn.edu

EDGAR DOBRIBAN
Department of Statistics and Data Science, the Wharton School, University of Pennsylvania, Philadelphia, PA, USA
dobriban@wharton.upenn.edu

ERIC TCHETGEN TCHETGEN
Department of Statistics and Data Science, the Wharton School, University of Pennsylvania, Philadelphia, PA, USA
ett@wharton.upenn.edu

1. DETAILS FOR TWO-SAMPLE TESTING

1.1. *Permutation test*

Permutation tests are a tool for assessing whether two distributions are equal or, more generally, whether two datasets are exchangeable. Given a test statistic $T : \mathcal{X}^{n+m} \to \mathbb{R}$, the p-value of the permutation test is

$$p = \frac{\sum_{\sigma \in \mathcal{S}_{n+m}} \mathbb{1}\left\{T(X_{\sigma(1)}, X_{\sigma(2)}, \dots, X_{\sigma(n+m)}) \geq T(X_1, X_2, \dots, X_{n+m})\right\}}{(n+m)!}. \tag{1}$$

The test statistic T can be any function—popular choices include the mean difference $T(x_1, \dots, x_{n+m}) = |\frac{1}{m}\sum_{j=1}^m x_{n+j} - \frac{1}{n}\sum_{i=1}^n x_i|$ and the quantile difference $T(x_1, \dots, x_{n+m}) = |Q_\tau(\{x_{n+1}, \dots, x_{n+m}\}) - Q_\tau(\{x_1, \dots, x_n\})$.

In practice, directly computing the p-value based on all $(n+m)!$ permutations is often computationally infeasible. Therefore, the following randomized version of the p-value is commonly used [Dwass, 1957, Lehmann and Romano, 2022]:

$$p = \frac{1 + \sum_{l=1}^L \mathbb{1}\left\{T(X_{\sigma_l(1)}, X_{\sigma_l(2)}, \dots, X_{\sigma_l(n+m)}) \geq T(X_1, X_2, \dots, X_{n+m})\right\}}{L+1}, \tag{2}$$

where $\{\sigma_1, \dots, \sigma_L\}$ is a random sample of permutations drawn uniformly from $\mathcal{S}_{n+m}$. Both the statistics (1) and (2) are valid in a distribution-free sense—i.e., under any distribution P, the inequality $\mathbb{P}\{p \leq \alpha\} \leq \alpha$ holds for any $\alpha \in (0,1)$ under the null hypothesis H_0 in (4), see, for example, Lehmann [2012].

1.2. *Rank-sum test and other methods*

If $\mathcal{X} \subset \mathbb{R}$, the rank-sum test [Mann and Whitney, 1947] uses a rank-based test statistic, $U = nm + n(n+1)/2 - R$, where R is the sum of ranks of the test observations $X_{n+1}, \dots, X_{n+m}$ within

© 2025 Biometrika Trust

the set of all observations $X_1, \dots, X_{n+m}$. If $\mathcal{X}$ is not a fully ordered set, each observation can first be mapped to a real number before applying the procedure. For small sample sizes n and m, the cumulative distribution function F_U of U can be computed directly to obtain a p-value, $F_U(U)$, with exact finite-sample validity. However, for large sample sizes, a normal approximation with an asymptotic guarantee is often used [Lehmann and D'Abrera, 2006]. Extensions include Rosenbaum [2005] and Biswas et al. [2014], among many others.

2. Testing based on multiple quantiles

The testing procedure based on the batch conformal p-value in (5) rejects the null hypothesis when a specified quantile of the test scores is unusually large compared to the reference scores. Consequently, if the distribution of the median does not change strongly under the alternative—e.g., it mainly changes in the tails—then the test's power might be low. If it is unknown where the shift is likely to occur, one might consider using multiple quantiles, such as the three quartiles, to capture the shift in an unknown region. Can we construct a valid p-value based on multiple quantiles of the test scores? We provide a positive answer next.

For clarity and intuition, we present a methodology for testing with two quantiles—generalization beyond two is straightforward. Given $0 < \eta_1 < \eta_2 < 1$, suppose we aim to reject the null if either $S^{\text{test}}_{(\eta_1)}$ or $S^{\text{test}}_{(\eta_2)}$ is large compared to the reference scores. Let $\tilde{\eta}_1 = \text{round}(\eta_1 \cdot \frac{n}{m})$ and $\tilde{\eta}_2 = \text{round}(\eta_2 \cdot \frac{n}{m})$ be the corresponding scaled ranks in the reference dataset, where round$(\cdot)$ rounds real numbers to the nearest integer, with round$(j + 1/2) = j$ for any integer j. We will design a test that rejects the null if either (1) $S^{\text{test}}_{(\eta_1)}$ is large compared to $S_{(\tilde{\eta}_1)}$ or (2) $S^{\text{test}}_{(\eta_2)}$ is large compared to $S_{(\tilde{\eta}_2)}$.

We can construct the p-value as

$$
\begin{aligned}
p &= \sum_{t=-\tilde{\eta}_1+1}^{n-\tilde{\eta}_2+1} w_t \cdot \mathbb{1}\left\{S^{\text{test}}_{(\eta_1)} \le S_{(\tilde{\eta}_1+t)}, S^{\text{test}}_{(\eta_2)} \le S_{(\tilde{\eta}_2+t)}\right\}, \text{ where} \\
w_t &= \sum_{\substack{t_1,t_2 \,:\, \max\{t_1,t_2\}=t \\ 1 \le \eta_1+\tilde{\eta}_1+t_1+1 < \eta_2+\tilde{\eta}_2+t_2+1 \le n+m}} \frac{\binom{\eta_1+\tilde{\eta}_1+t_1-2}{\eta_1-1}\binom{\eta_2+\tilde{\eta}_2+t_2-\eta_1+\tilde{\eta}_1+t_1-1}{\eta_2-\eta_1-1}\binom{n+m-\eta_2-\tilde{\eta}_2-t_2+1}{m-\eta_2}}{\binom{n+m}{m}}.
\end{aligned}
\tag{3}
$$

Here, we define $S_{(n+1)} = +\infty$. Observe that the p above tends to become small when either $S^{\text{test}}_{(\eta_1)}$ or $S^{\text{test}}_{(\eta_2)}$ is large. The following result shows the validity of this method.

THEOREM 1. *The statistic p in* (3) *is a valid p-value for the null hypothesis H_0 in* (4).

The proof is presented in Section 6.4.

3. Additional experimental results

3.1. *Comparison with permutation-test-based method*

Here, we repeat the simulation in Section 3.2 with additional baseline methods. We fix the score to score A and compare the following three methods:

1. the Benjamini–Hochberg procedure applied to the batch conformal p-values (proposed procedure),
2. the Benjamini–Hochberg procedure applied to the permutation test p-values (no theoretical guarantee), and

3. the Benjamini–Yekutieli procedure applied to the permutation test p-values.

The results are shown in Figure 2. As discussed in Section 2.2, the Benjamini–Yekutieli procedure provides conservative detection with low power. The Benjamini–Hochberg procedure applied to the permutation test p-values shows results comparable to those of the proposed procedure—although it has no theoretical guarantee and is computationally much heavier. In this setting, the proposed procedure can be viewed as a 'correction' of this method that enables provable false discovery rate control while being computationally cheaper.

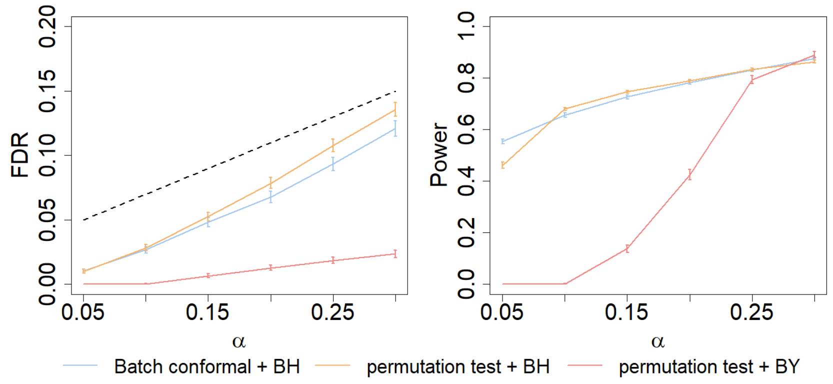

Fig. 1. False discovery rate and power of the proposed procedure and the permutation-test-based methods.

3.2. *False discovery proportion and true positive proportion*

Here, we present the plots of the false discovery proportion and the true positive proportion. Figure 2 shows the results from the simulation in Section 3.2 with score A. Note that the experiment was performed under small group number, and thus the false discovery proportion and the true positive proportion are highly variable—still, the false discovery proportion values are mostly below the level α.

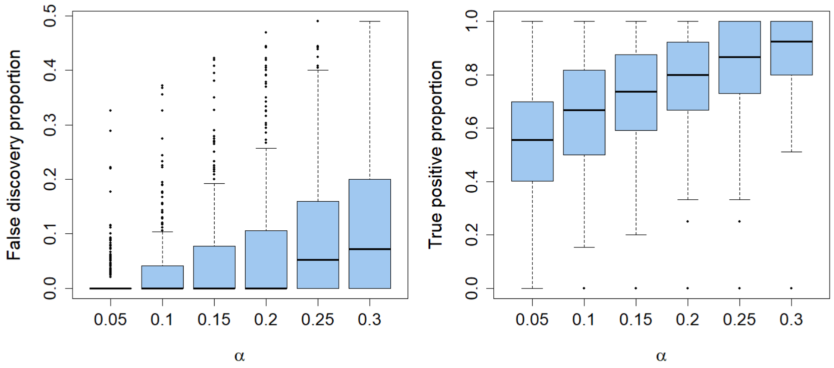

Fig. 2. False discovery proportion and true positive proportion of the proposed procedure

3.3. *Two sample test under shift in the tail*

We provide additional experimental results that supplement the experiment in Section 3.3, to illustrate the usefulness of the batch conformal p-value in the setting where the shift occurs in the tail. We generate two samples from the following distributions:

$$(1)\ X \sim \mathcal{N}(0,1), \qquad (2)\ X \sim 0.9 \cdot \mathcal{N}(0,1) + 0.1 \cdot \delta_5,$$

where δ_5 denotes the point mass at 5. In addition to the methods included in the experiment in Section 3.3—with $q = 0.9$ for the batch conformal p-value and the permutation test—, we further compare with methods from Gretton et al. [2012], which introduced the kernel two-sample test based on the maximum mean discrepancy (MMD) statistic, and from Ramdas et al. [2017], which proposed a method based on the Wasserstein distance. We explore small-sample settings where distribution-free methods are typically preferred: (i) $n = m = 30$, and (ii) $n = 50$, $m = 20$, where n and m denote the sample sizes of the data from distributions (1) and (2), respectively. The results are shown in Figure 3, illustrating that the batch conformal p-value, with an appropriate choice of the quantile-based test statistic, achieves higher power compared to methods based on MMD or the Wasserstein distance.

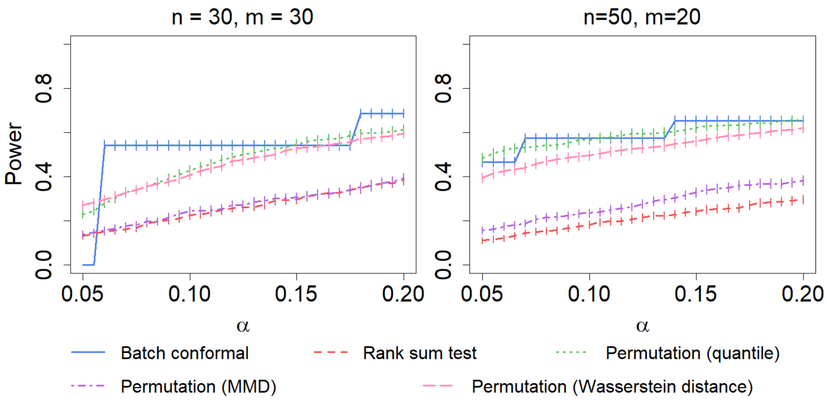

Fig. 3. Power of different two sample testing procedures, under two settings of sample sizes.

3.4. *Data-driven choice of the test statistic*

In this section, we empirically explore the method for selecting the test statistic or hyperparameter discussed in Section 2.3. For evaluation purposes, we consider the following three simple settings:

1. Setting 1: Null $N(0,3)$ vs Alternative $N(3,3)$.
2. Setting 2: Null $N(0,1)$ vs Alternative $N(0,3)$.
3. Setting 3: Null $N(0,2)$ vs Alternative $N(1,3)$.

The first setting reflects a case in which the default choice of the median is already appropriate, in the sense that it is likely to successfully detect the shift. The second setting corresponds to a shift in scale but not in location, and thus the default choice is not appropriate. The third setting reflects a distributional shift in both location and scale.

We generate a reference dataset of size 200 from the null distribution, along with 50 groups of comparison data, each of size 100, with half of the groups drawn from the null distribution and the other half from the alternative distribution. We compare two methods: (1) the proposed

procedure with the median as the test statistic, and (2) the proposed procedure with a data-driven choice of the test statistic. For the second method, we split each reference and comparison dataset into two subsets of equal size. Using one split, we compute the $0.1, 0.2, \ldots, 0.9$-sample quantiles of the scores. Then, for each comparison group, we identify the quantile that shows the largest difference from the reference group and set the corresponding quantile as η_k. The results are shown in Figure 4.

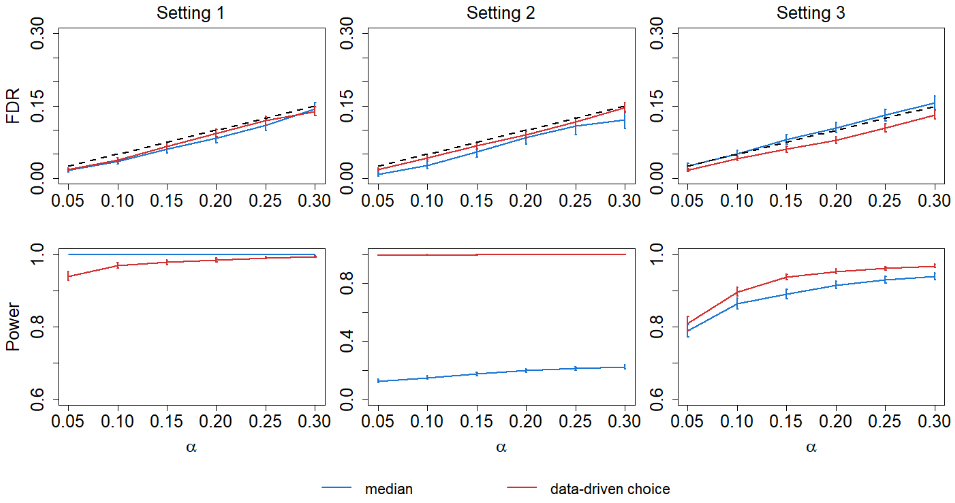

Fig. 4. False discovery rate and power of the proposed procedure with the median statistic and the data-driven statistic.

We observe that in Setting 1, where the median is already an appropriate statistic that achieves power one, the data-driven choice of the test statistic also attains high power and incurs only a small loss due to data splitting. In Setting 2, the data-driven method achieves substantially higher power, as expected. In the less extreme Setting 3, we also observe an improvement from the data-driven choice of the test statistic. To summarize, the data-driven choice of the test statistic can be helpful in practical settings where the true distributions are unknown, and the data splitting required by this method does not lead to a substantial loss of power compared to the default approach.

3.5. *Results under additional settings*

In this section, we present simulation results under additional settings for the feature dimension and the group sizes. First, we repeat the steps in Section 3.2 with score A, but vary the dimension of the variable X, taking $p = 5, 10$, and 20. The results are shown in Figure 5, illustrating that the false discovery rate is controlled at the theoretical bound across these different settings.

Next, we vary the sizes of the comparison groups. We generate the group sizes in three ways:

1. Setting 1: $n_k \overset{\text{i.i.d.}}{\sim} 1 + \text{Poisson}(10)$,
2. Setting 2: $n_k \overset{\text{i.i.d.}}{\sim} 5 + \text{Poisson}(20)$,
3. Setting 3: $n_k \equiv 10$.

The results are shown in Figure 6, again illustrating that the procedure controls the false discovery rate at different levels.

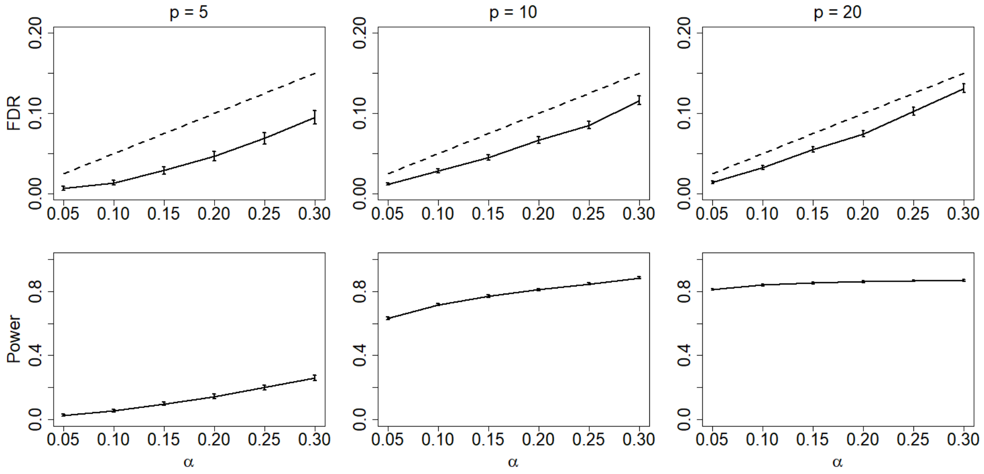

Fig. 5. False discovery rate and power of the proposed procedure under different dimension of the side information.

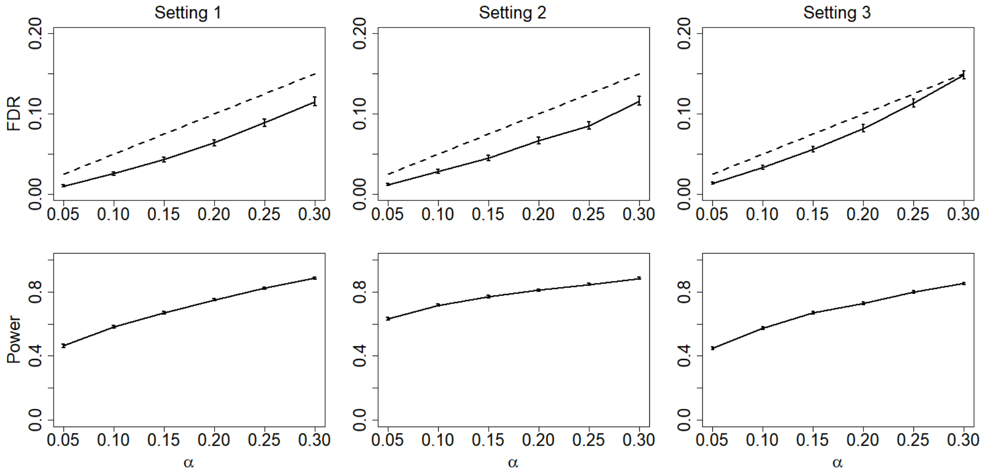

Fig. 6. False discovery rate and power of the proposed procedure under different sizes of the comparison groups

4. POWER IN A SIMPLE SCORE-LEVEL MODEL

In this subsection, we further illustrate what kinds of alternatives are detected by the proposed procedure. We work directly at the level of the scores, on which our method operates. We first derive an exact finite-sample rejection probability for a two sample test based on the batch conformal p-value under an arbitrary continuous alternative score distribution. We then pass to a large-reference idealization, in which the resulting non-null p-value cdf can be written explicitly. Finally, we embed this explicit non-null CDF into the classical independent two-groups asymptotic theory of the Benjamini–Hochberg procedure.

Relation to the literature. The independent two-groups model is standard in large-scale multiple testing; see, for instance, Genovese and Wasserman [2004], Efron [2004, 2007, 2008]. The asymptotic behavior of the Benjamini–Hochberg procedure under independence is classical.

Genovese and Wasserman [2002] study the asymptotic "deciding point" and the limiting rejection threshold. Genovese and Wasserman [2004] recast the procedure in threshold form using the empirical cdf of the p-values. Chi [2007] identifies the associated critical value and studies the resulting criticality phenomenon. Neuvial [2008, 2013] formulate the asymptotic threshold as the rightmost crossing $\sup\{t : G(t) \geq t/\alpha\}$ and prove almost sure convergence of the empirical Benjamini–Hochberg threshold under independence. Ferreira and Zwinderman [2006b,a] study asymptotic power, sample-size, and threshold properties, while Izmirlian [2020] proves strong consistency and asymptotic normality for several Benjamini–Hochberg-related quantities, including power-type quantities. Exact finite-sample average power calculations for the Benjamini–Hochberg procedure are given by Glueck et al. [2008].

In our setting, the cdf of the non-null p-value can be written explicitly from the construction of our p-value. For simplicity, we begin with the two-sample setting. Let $S_1, \ldots, S_n$ denote the reference scores and let $T_1, \ldots, T_m$ denote the comparison scores. We assume that $S_1, \ldots, S_n$ are i.i.d. with a continuous cdf F_0, that $T_1, \ldots, T_m$ are i.i.d. with a continuous cdf F_1, and that the two samples are independent. Let $T_{(\eta)}$ denote the η-th order statistic of the comparison scores, where $\eta \in [m]$ is fixed.

Recall that the batch conformal p-value from (5) can be written as $p = \sum_{i=1}^{n} w_i \mathbb{1}\left\{T_{(\eta)} \leq S_{(i)}\right\} + w_{n+1}$, where

$$w_i = \frac{\binom{i+\eta-2}{\eta-1}\binom{n+m-i-\eta+1}{m-\eta}}{\binom{n+m}{m}}, \qquad i \in [n+1].$$

Now define for each $\alpha \in (0, 1)$,

$$r_\alpha = \min\left(\left\{r \in [n+1] : \sum_{i=r}^{n+1} w_i \leq \alpha\right\} \cup \{n+2\}\right).$$

Then the level-α test based on the batch conformal p-value rejects if and only if $p \leq \alpha$, equivalently if and only if the rank $R = 1 + \sum_{i=1}^{n} \mathbb{1}\left\{S_i < T_{(\eta)}\right\}$ satisfies $R \geq r_\alpha$.

PROPOSITION 1 (EXACT FINITE-SAMPLE REJECTION PROBABILITY). *For every $\alpha \in (0, 1)$,*

$$\mathbb{P}_{F_0,F_1}\{p \leq \alpha\} = \int \sum_{j=r_\alpha-1}^{n} \binom{n}{j} F_0(x)^j \left(1 - F_0(x)\right)^{n-j} dF_{1,(\eta)}(x),$$

where $F_{1,(\eta)}$ denotes the cdf of $T_{(\eta)}$. Equivalently, if $U \sim \mathrm{Beta}(\eta, m-\eta+1)$, then

$$\mathbb{P}_{F_0,F_1}\{p \leq \alpha\} = \mathbb{E}\left[\sum_{j=r_\alpha-1}^{n} \binom{n}{j} q(U)^j \left(1 - q(U)\right)^{n-j}\right], \qquad q(U) = F_0(F_1^{-1}(U)).$$

In particular, the rejection probability depends on the alternative only through the distribution of $F_0(T_{(\eta)})$.

Proposition 1 makes the detection mechanism explicit: the power is governed by the random variable $F_0(T_{(\eta)})$, that is, by how large the selected comparison quantile is when measured on the reference scale.

We now move to a large-reference idealization. In that idealization, the comparison with the empirical reference sample is replaced by comparison with the reference cdf F_0. The cdf of the non-null p-value can then be written explicitly. We work in a location family where the

distribution of the alternative scores is a shifted version of the distribution of the null scores, i.e., $F_1(x) = F_0(x - \mu)$ for all x. We prove the following result.

COROLLARY 1 (LARGE-REFERENCE IDEALIZATION AND EXPLICIT CDF OF NON-NULL p-VALUE). *Assume that* $F_1(x) = F_0(x - \mu)$ *for some* $\mu > 0$. *Let* $B_{\eta,m-\eta+1}$ *denote the cdf of the* $\mathrm{Beta}(\eta, m - \eta + 1)$ *distribution, and let* $b_t = B^{-1}_{\eta,m-\eta+1}(1 - t)$ *for* $t \in (0, 1)$. *Define the idealized batch conformal p-value by* $p_\infty = 1 - B_{\eta,m-\eta+1}\big(F_0(T_{(\eta)})\big)$. *Then under the null* $F_1 = F_0$, *the random variable* p_∞ *is uniformly distributed on* $[0, 1]$. *Under the alternative* $F_1(x) = F_0(x - \mu)$, *its cdf is*

$$H_{\mu,\eta}(t) = 1 - B_{\eta,m-\eta+1}\big(F_0(F_0^{-1}(b_t) - \mu)\big), \qquad t \in (0, 1).$$

Moreover, for each fixed $t \in (0, 1)$, *the quantity* $H_{\mu,\eta}(t)$ *is nondecreasing in* μ.

Remark 1 (*Which alternatives are detected?*). Here, we discuss the role of the parameter η. The distribution $\mathrm{Beta}(\eta, m - \eta + 1)$ has mean $\eta/(m + 1)$ and concentrates around that value when m is moderately large. Consequently, the method primarily probes the behavior of the difference $F_1^{-1}(u) - F_0^{-1}(u)$ for u near $\eta/(m + 1)$. Thus, choosing $\eta \approx 0.5m$ targets median shifts, whereas choosing $\eta \approx 0.9m$ targets upper-tail shifts. Conversely, if F_0 and F_1 differ mainly in regions far from the chosen quantile level, then the power can remain low even though the two distributions are different.

We now turn to the multiple-testing problem. By leveraging the classical asymptotic theory of the Benjamini-Hochberg procedure [Genovese and Wasserman, 2002, 2004, Chi, 2007, Neuvial, 2008, 2013, Ferreira and Zwinderman, 2006a, Izmirlian, 2020], we can characterize the power of our procedure in the following large-reference idealization. Suppose each comparison group has size m and uses the same rank $\eta \in [m]$. Let F_0 denote the score distribution under the null, and suppose that under the alternative the score distribution is the one-sided location shift $F_1(x) = F_0(x - \mu)$ with $\mu > 0$. In the large-reference limit, the batch conformal p-value for one group becomes

$$p_\infty = 1 - B_{\eta,m-\eta+1}\big(F_0(T_{(\eta)})\big),$$

where $T_{(\eta)}$ is the ηth order statistic of the m comparison scores and $B_{\eta,m-\eta+1}$ denotes the cdf of the $\mathrm{Beta}(\eta, m - \eta + 1)$ distribution. Under the null, p_∞ is uniformly distributed on $[0, 1]$, while under the alternative its cdf is

$$H_{\mu,\eta}(t) = 1 - B_{\eta,m-\eta+1}\big(F_0(F_0^{-1}(b_t) - \mu)\big), \qquad b_t = B^{-1}_{\eta,m-\eta+1}(1 - t).$$

Now consider an independent two-groups model in which a proportion π_0 of the hypotheses are null and a proportion $\pi_1 = 1 - \pi_0$ are non-null, so that the marginal cdf of the input p-values is $G_{\mu,\eta}(t) = \pi_0 t + \pi_1 H_{\mu,\eta}(t)$. By classical results on the asymptotics of the Benjamini–Hochberg procedure under independent mixture models [Genovese and Wasserman, 2002, 2004, Chi, 2007, Neuvial, 2008, 2013, Ferreira and Zwinderman, 2006a, Izmirlian, 2020], the empirical Benjamini–Hochberg threshold converges to the rightmost crossing of $G_{\mu,\eta}(t)$ with t/α. This yields the following corollary.

COROLLARY 2. *Assume the one-sided location-shift model* $F_1(x) = F_0(x - \mu)$, *and define*

$$t_\star = \sup\{t \in [0, 1] : \pi_0 t + \pi_1 H_{\mu,\eta}(t) \geq t/\alpha\}.$$

Assume that $t_\star \in (0, 1)$ *is isolated, in the sense that there exists* $\delta_0 > 0$ *such that* $\pi_0 t + \pi_1 H_{\mu,\eta}(t) > t/\alpha$ *for all* $t \in (t_\star - \delta_0, t_\star)$ *and* $\pi_0 t + \pi_1 H_{\mu,\eta}(t) < t/\alpha$ *for all* $t \in (t_\star, t_\star + \delta_0)$. *Then the asymp-*

totic power of the Benjamini-Hochberg procedure is given as

$$\mathrm{Pow}_{\mathrm{BH}} = H_{\mu,\eta}(t_\star) = 1 - B_{\eta,m-\eta+1}\big(F_0(F_0^{-1}(b_{t_\star}) - \mu)\big), \qquad b_{t_\star} = B^{-1}_{\eta,m-\eta+1}(1 - t_\star).$$

Remark 2 (*Interpretation*). The corollary shows that, in the large-reference idealization, the power of the proposed procedure is completely determined by two quantities: the non-null p-value cdf $H_{\mu,\eta}$ and the asymptotic Benjamini-Hochberg cutoff $t_\star$. Specifically, $\mathrm{Pow}_{\mathrm{BH}} = H_{\mu,\eta}(t_\star)$ is the probability that a non-null idealized batch conformal p-value falls below the limiting Benjamini-Hochberg threshold. The threshold $t_\star$ itself is the rightmost crossing of the mixture cdf $\pi_0 t + \pi_1 H_{\mu,\eta}(t)$ with the line t/α, and therefore captures the effect of multiplicity through α and the signal sparsity through π_0 and π_1.

The formula also clarifies the role of the hyperparameter η: it determines which quantile of the comparison-score distribution is being tested. Thus $\eta \approx m/2$ emphasizes central shifts, whereas larger values of η place more emphasis on upper-tail behavior. Consequently, the procedure is most powerful when the chosen score quantile under the alternative is substantially larger than under the null.

Remark 3 (*Consistency under favorable alternatives*). Here, we show that in the same large-reference idealization, the procedure is consistent against favorable one-sided location shifts when the group size grows.

Specifically, let $\eta = \eta_m$ depend on m in such a way that $\eta_m/m \to \tau \in (0, 1)$, and write

$$H^{(m)}_{\mu,\eta_m}(t) = 1 - B_{\eta_m,m-\eta_m+1}\big(F_0(F_0^{-1}(b_t^{(m)}) - \mu)\big), \qquad b_t^{(m)} = B^{-1}_{\eta_m,m-\eta_m+1}(1 - t).$$

Assume that F_0 is continuous and strictly increasing, and fix $\mu > 0$. Then, for every fixed $t \in (0, 1)$,

$$H^{(m)}_{\mu,\eta_m}(t) \to 1 \qquad \text{as } m \to \infty.$$

Hence the idealized single-test power tends to 1 at every fixed level.

Moreover, if

$$t_\star^{(m)} = \sup\{t \in [0, 1] : \pi_0 t + \pi_1 H^{(m)}_{\mu,\eta_m}(t) \geq t/\alpha\},$$

then the corresponding asymptotic power of the Benjamini-Hochberg also tends to 1:

$$\mathrm{Pow}^{(m)}_{\mathrm{BH}} = H^{(m)}_{\mu,\eta_m}(t_\star^{(m)}) \to 1 \qquad \text{as } m \to \infty.$$

Proof. Let $U_m \sim \mathrm{Beta}(\eta_m, m - \eta_m + 1)$. Since

$$\mathbb{E}[U_m] = \eta_m/(m + 1) \to \tau \text{ and } \mathrm{Var}(U_m) = \frac{\eta_m(m - \eta_m + 1)}{(m + 1)^2(m + 2)} \to 0 \qquad \text{as } m \to \infty,$$

we have $U_m \to \tau$ in probability. Therefore, the $(1 - t)$-quantile $b_t^{(m)}$ of the $\mathrm{Beta}(\eta_m, m - \eta_m + 1)$ distribution also converges to τ for every fixed $t \in (0, 1)$.

Since F_0 is continuous and strictly increasing,

$$q_t^{(m)} := F_0(F_0^{-1}(b_t^{(m)}) - \mu) \to F_0(F_0^{-1}(\tau) - \mu) < \tau \qquad \text{as } m \to \infty.$$

It follows that there exists a constant $\delta > 0$ such that $q_t^{(m)} \leq \tau - \delta$ holds for all sufficiently large m. Therefore, as m tends to infinity,

$$1 - H^{(m)}_{\mu,\eta_m}(t) = B_{\eta_m,m-\eta_m+1}(q_t^{(m)}) = \mathbb{P}(U_m \leq q_t^{(m)}) \leq \mathbb{P}(U_m \leq \tau - \delta) \to 0,$$

which implies $H^{(m)}_{\mu,\eta_m}(t) \to 1$.

For the second claim, fix any constant c such that $0 < c < \alpha\pi_1/(1-\alpha\pi_0)$. Then $\pi_0 c + \pi_1 > c/\alpha$. Since $H^{(m)}_{\mu,\eta_m}(c) \to 1$, we have $\pi_0 c + \pi_1 H^{(m)}_{\mu,\eta_m}(c) > c/\alpha$ for all sufficiently large m, which implies $t^{(m)}_\star \geq c$. since $H^{(m)}_{\mu,\eta_m}$ is nondecreasing,

$$\mathrm{Pow}^{(m)}_{\mathrm{BH}} = H^{(m)}_{\mu,\eta_m}(t^{(m)}_\star) \geq H^{(m)}_{\mu,\eta_m}(c) \to 1.$$

and this implies $\mathrm{Pow}^{(m)}_{\mathrm{BH}} \to 1$, since $\mathrm{Pow}^{(m)}_{\mathrm{BH}} \leq$ holds deterministically. □

5. Special case: distribution-free two-sample test

Here, we briefly discuss a special case of the setting introduced in Section 1.2: having only one comparison dataset, which corresponds to two-sample testing. Suppose we have two datasets, the reference dataset $X_1, \ldots, X_n \overset{\text{i.i.d.}}{\sim} P$ and the comparison dataset $X_{n+1}, \ldots, X_{n+m} \overset{\text{i.i.d.}}{\sim} Q$, and aim to test the null hypothesis

$$H_0 : P = Q. \tag{4}$$

This is clearly a special case of the problem introduced in Section 1.2, where $K = 1$, $P^{(1)} = Q$, and $n_1 = m$. In this two-sample problem, there is no need for multiple testing. Instead, the problem reduces to constructing a test $\phi : \mathcal{X}^n \times \mathcal{X}^m \to \{0, 1\}$ with finite-sample type I error control

$$\begin{aligned}&\mathbb{E}_{H_0}\left[\phi((X_1,\ldots,X_n),(X_{n+1},\ldots,X_{n+m}))\right]\\&\qquad = \mathbb{E}_{X_1,\ldots,X_{n+m}\overset{\text{i.i.d.}}{\sim}P}\left[\phi((X_1,\ldots,X_n),(X_{n+1},\ldots,X_{n+m}))\right] \leq \alpha\end{aligned}$$

for a predefined level $\alpha \in (0, 1)$, under any distribution P. Well-known methods that achieve this goal include the permutation test [Eden and Yates, 1933, Fisher, 1935, Dwass, 1957] and the rank-sum test Mann and Whitney [1947]. We briefly review these methods in Section 1 of the Supplementary Material: the permutation test can have a high computational cost or requires randomization to avoid this, while the rank-sum test is usually used with its asymptotic normal approximation.

We now discuss the proposed batch conformal p-value, which is computationally cheaper and non-randomized. With the score function $s : \mathcal{X} \to \mathbb{R}$ chosen such that larger scores are more likely to occur under Q when $P \neq Q$, define $S_i = s(X_i)$ for $i \in [n+m]$. Now, let $S_{(i)}$ denote the i-th order statistic of the *reference scores* $S_1, \ldots, S_n$, and $S^{\text{test}}_{(\eta)}$ the η-th order statistic of the *comparison scores* $S_{n+1}, \ldots, S_{n+m}$, for a predetermined $\eta \in [m]$. In this case, the batch conformal p-value for testing $P = Q$ from (2) becomes

$$p = \sum_{i=1}^{n} \frac{\binom{i+\eta-2}{\eta-1}\binom{n+m-i-\eta+1}{m-\eta}}{\binom{n+m}{m}} \cdot \mathbb{1}\left\{S^{\text{test}}_{(\eta)} \leq S_{(i)}\right\} + \frac{\binom{n+\eta-1}{\eta-1}}{\binom{n+m}{m}}. \tag{5}$$

As a consequence of the validity of batch conformal p-values, if the scores $S_1, \ldots, S_{n+m}$ are exchangeable, then the statistic p in (5) is a valid p-value for the null hypothesis H_0 in (4).

Remark 4. If the score distribution has point masses, we apply uniform tie-breaking to determine the order of scores. Another standard approach is to add a small amount of noise to the score [Kuchibhotla, 2020], so that the resulting adjusted score has a nonatomic distribution. For example, one can construct the p-value using the scores $\tilde{S}_i = S_i + \varepsilon_i$, where $\varepsilon_i \overset{\text{i.i.d.}}{\sim} \mathcal{N}(0, 10^{-10})$. While the validity of batch conformal p-values holds with the uniform tie-breaking strategy without introducing additional noise, the tie-breaking-with-noise strategy can be useful, particularly with multiple comparison groups—the main focus of this work—followed by multiple p-values reusing

the same reference dataset, where "uniform tie-breaking" may result in a lack of "consistency" across groups, thereby requiring the almost-sure distinctness condition in Theorem 1.

By the validity of batch conformal p-values, the test $\phi = \mathbb{1}\{p \leq \alpha\}$ controls the type I error at level α, for any $\alpha \in (0, 1)$. Intuitively, this test rejects the null if the η-sample quantile of the test scores is unusually large compared to the reference scores. In Supplementary Material, we present a generalized version of the p-value from (5) that depends on multiple quantiles.

While our main focus is on the setting of multiple comparison groups, the batch conformal p-value can have benefits over well-known methods in some scenarios. First, the goal of our method is to detect changes in the *quantiles* of the distributions, and thus can be used to detect changes in the tails (e.g., the new treatment works better for the top 10% patients with the highest blood pressure). In contrast, the popular rank-sum test aims to detect changes in the "bulk" of the distribution. This allows our method to detect a distinct and complementary kind of effects; see the illustration in Section 3.3.

Moreover, our method also has an advantage compared to permutation tests. While one can use these with the same quantile test statistic as our method (and thus in principle they can detect the same effects), a key limitation is that permutation tests can be computationally heavy, requiring the re-computation of test statistics over many permutations. While one can randomly sample permutations [Lehmann and Romano, 2022], one may still need to sample a large number to get significant results (at least $\lceil 1/\alpha \rceil - 1$ to have the possibility of a p-value less than or equal to α). This is especially severe in situations where there is *multiplicity*, i.e., when multiple tests are performed, and where higher levels of significance are required due to multiplicity adjustments. For instance, in genomics, permutation tests are performed often, but are known to be expensive [see e.g., Stranger et al., 2007, Salojärvi et al., 2017, John et al., 2022, etc]. Our methods could lead to significant computational savings in such situations.

6. Proofs of theorems

6.1. *Supporting lemmas*

We first introduce supporting lemmas for the validity of batch conformal p-values and Theorem 1, establishing properties of the distribution of the ranks of test scores.

Lemma 1 (Lee et al. [2024]). *For positive integers $n, m \in \mathbb{N}$, let $S_1, S_2, \ldots, S_{n+m} \sim P_S$ be exchangeable random variables, which are distinct almost surely. Let $\bar{S}_\uparrow = (S_{(1)}, S_{(2)}, \ldots, S_{(n+m)})$ be the vector of their order statistics. For $j \in [m]$, let $T_j = \sum_{k=1}^{n+m} \mathbb{1}\{S_{(k)} \leq S_{n+j}\}$ denote the rank of S_{n+j} among the $n+m$ scores in increasing order, and let $R_1 < \ldots < R_m$ denote the ordered ranks obtained by rearranging $T_1, \ldots, T_m$. Then,*

$$(R_1, R_2, \ldots, R_m) \mid \bar{S}_\uparrow \sim \mathrm{Unif}(\{(r_1, \ldots, r_m) : 1 \leq r_1 < \ldots < r_m \leq n+m\}). \tag{6}$$

We omit the proof of Lemma 1 as it is a direct consequence of the exchangeability condition and is also established in Lee et al. [2024].[1]

Lemma 2 (Stochastic order relations between ranks). *If the random variables $R_1, \ldots, R_m$ follow the distribution* (6)*, then:*

1. *For any $t \in [m]$, $(R_1, \ldots, R_{t-1}) \perp\!\!\!\perp (R_{t+1}, \ldots, R_m) \mid (R_t, \bar{S}_\uparrow)$.*

[1] Lee et al. [2024] derives that the marginal distribution of the vector $(R_1, \ldots, R_m)$ is uniform from the exchangeability condition. However, since conditioning on $\bar{S}_\uparrow$—or equivalently, conditioning on the set of scores—does not break the exchangeability, the same steps hold for the conditional distribution of $(R_1, \ldots, R_m)$ given $\bar{S}_\uparrow$.

2. *For any $t \in [m]$, $r \in [n+m]$ and $1 \leq q \leq q' \leq n+m$, the following inequalities hold:*

$$\mathbb{P}\left\{R_{t-1} \leq r \;\middle|\; R_t = q, \bar{S}_\uparrow\right\} \geq \mathbb{P}\left\{R_{t-1} \leq r \;\middle|\; R_t = q', \bar{S}_\uparrow\right\}.$$
$$\mathbb{P}\left\{R_{t+1} \geq r \;\middle|\; R_t = q, \bar{S}_\uparrow\right\} \leq \mathbb{P}\left\{R_{t+1} \geq r \;\middle|\; R_t = q', \bar{S}_\uparrow\right\}.$$

Proof Proof of Lemma 2. **Proof of the first claim.** Since the ranks $R_1, \ldots, R_m$ are independent of $\bar{S}_\uparrow$ by Lemma 1, it is sufficient to prove that $(R_1, \ldots, R_{t-1})$ and $(R_{t+1}, \ldots, R_m)$ are conditionally independent given R_t. Let $L = |\{(r_1, \ldots, r_m) : 1 \leq r_1 < \ldots < r_m \leq n+m\}| = \binom{n+m}{m}$. Next, fix any $1 \leq r_1 < \ldots < r_m \leq n+m$, and compute

$$\mathbb{P}\{R_1 = r_1, \ldots, R_{t-1} = r_{t-1} \mid R_t = r_t\} = \frac{\mathbb{P}\{R_1 = r_1, \ldots, R_{t-1} = r_{t-1}, R_t = r_t\}}{\mathbb{P}\{R_t = r_t\}}$$
$$= \frac{\sum_{r_t < v_{t+1} < \ldots < v_m \leq n+m} \mathbb{P}\{R_1 = r_1, \ldots, R_{t-1} = r_{t-1}, R_t = r_t, R_{t+1} = v_{t+1}, \ldots, R_m = v_m\}}{\sum_{1 < u_1 < \ldots < u_{t-1} < r_t < v_{t+1} < \ldots < v_m \leq n+m} \mathbb{P}\{R_1 = u_1, \ldots, R_{t-1} = u_{t-1}, R_t = r_t, R_{t+1} = v_{t+1}, \ldots, R_m = v_m\}}$$
$$= \frac{\frac{1}{L} \cdot |\{(v_{t+1}, \ldots, v_m) : r_t < v_{t+1} < \ldots < v_m \leq n+m\}|}{\frac{1}{L} \cdot |\{(u_1, \ldots, u_{t-1}, v_{t+1}, \ldots, v_m) : 1 \leq u_1 < \ldots < u_{t-1} < r_t < v_{t+1} < \ldots < v_m \leq n+m\}|}$$
$$= \frac{\binom{n+m-r_t}{m-t}}{\binom{r_t-1}{t-1} \cdot \binom{n+m-r_t}{m-t}} = \frac{1}{\binom{r_t-1}{t-1}}.$$

Similarly, we have $\mathbb{P}\{R_{t+1} = r_{t+1}, \ldots, R_m = r_m \mid R_t = r_t\} = 1/\binom{n+m-r_t}{m-t}$. Observe that the calculation above also reveals that $\mathbb{P}\{R_t = r_t\} = \frac{1}{L} \cdot \binom{r_t-1}{t-1} \cdot \binom{n+m-r_t}{m-t}$. Thus, we have

$$\mathbb{P}\{R_1 = r_1, \ldots, R_{t-1} = r_{t-1}, R_{t+1} = r_{t+1}, \ldots, R_m = r_m \mid R_t = r_t\}$$
$$= \frac{\mathbb{P}\{R_1 = r_1, \ldots, R_{t-1} = r_{t-1}, R_t = r_t, R_{t+1} = r_{t+1}, \ldots, R_m = r_m\}}{\mathbb{P}\{R_t = r_t\}} = \frac{\frac{1}{L}}{\frac{1}{L} \cdot \binom{r_t-1}{t-1} \cdot \binom{n+m-r_t}{m-t}}$$
$$= \mathbb{P}\{R_1 = r_1, \ldots, R_{t-1} = r_{t-1} \mid R_t = r_t\} \cdot \mathbb{P}\{R_{t+1} = r_{t+1}, \ldots, R_m = r_m \mid R_t = r_t\}.$$

The equality above holds for any sequence $r_1 < \ldots < r_m$, thereby proving the claim.

Proof of the second claim. Fix $t \geq 2$. Define $f_q(l) = \mathbb{P}\{R_{t-1} = l, R_t = q\}$ for $1 \leq l < q \leq n+m$. Using similar calculations as before, we find that $f_q(l) = \frac{1}{L} \cdot \binom{l-1}{t-2} \cdot \binom{m+n-q}{m-t}$. Therefore,

$$\mathbb{P}\{R_{t-1} \leq r \mid R_t = q\} = \frac{\mathbb{P}\{R_{t-1} \leq r, R_t = q\}}{\mathbb{P}\{R_t = q\}} = \frac{\sum_{l=1}^{r} f_q(l)}{\sum_{l=1}^{q-1} f_q(l)} = \frac{\sum_{l=1}^{r} \binom{l-1}{t-2}}{\sum_{l=1}^{q-1} \binom{l-1}{t-2}} \geq \frac{\sum_{l=1}^{r} \binom{l-1}{t-2}}{\sum_{l=1}^{q'-1} \binom{l-1}{t-2}}$$
$$= \mathbb{P}\{R_{t-1} \leq r \mid R_t = q'\}.$$

The second inequality can be derived similarly.

6.2. *Proof of Marginal Validity of Batch Conformal P-values*

For simplicity, we use the notations for two-sample testing (Section 5), i.e., write $P^{(k)} = Q$, $n_k = m$, and $(S_1^{(k)}, \ldots, S_{n_k}^{(k)}) := (S_{n+1}, \ldots, S_{n+m})$. We first consider the case where the scores are all distinct almost surely. It is sufficient to prove that

$$p = \sum_{i=1}^{n} w_i \cdot \mathbb{1}\left\{S_{(\eta)}^{\text{test}} \leq S_{(i)}\right\} + w_{n+1}, \qquad \text{where } w_i = \frac{\binom{\eta+i-2}{\eta-1}\binom{n+m-\eta-i+1}{m-\eta}}{\binom{n+m}{m}}, i \in [n]$$

is super-uniform under H_0, for any n, m, and $0 \le \eta \le m$. Define $R_1 < \ldots < R_m$ as in Lemma 1. By the definition of R_η, there are $R_\eta - \eta$ number of reference scores that are smaller than $S^{\text{test}}_{(\eta)}$. Consequently, for any $i \in [n]$, the following equivalence holds:

$$S^{\text{test}}_{(\eta)} \le S_{(i)} \iff R_\eta - \eta + 1 \le i. \tag{7}$$

Now we find the distribution of $R_\eta - \eta + 1$. By Lemma 1, for any $i \in [n+1]$,

$$\begin{aligned}\mathbb{P}\left\{R_\eta - \eta + 1 = i\right\} &= \frac{1}{\binom{n+m}{m}} \cdot \left|\{(r_1, \ldots, r_m) : 1 \le r_1 < \ldots < r_m \le n+m, r_\eta = \eta + i - 1\}\right| \\ &= \frac{1}{\binom{n+m}{m}} \cdot \binom{\eta + i - 2}{\eta - 1}\binom{n + m - \eta - i + 1}{m - \eta} = w_i.\end{aligned}$$

It follows that the cumulative distribution function of $R_\eta - \eta + 1$ is $x \mapsto F_{R_\eta - \eta + 1}(x) = \sum_{i=1}^{n+1} w_i \mathbb{1}\{x \le i\}$. Therefore, for any $\alpha \in (0, 1)$,

$$\begin{aligned}\mathbb{P}\{p \le \alpha\} = \mathbb{P}\left\{\sum_{i=1}^{n} w_i \mathbb{1}\left\{S^{\text{test}}_{(\eta)} \le S_{(i)}\right\} + w_{n+1} \le \alpha\right\} &= \mathbb{P}\left\{\sum_{i=1}^{n+1} w_i \mathbb{1}\left\{R_\eta - \eta + 1 \le i\right\} \le \alpha\right\} \\ &= \mathbb{P}\left\{F_{R_\eta - \eta + 1}(R_\eta - \eta + 1) \le \alpha\right\} \le \alpha, \end{aligned} \tag{8}$$

as desired.

Now, suppose ties exist among the scores, and we apply uniform tie-breaking to determine the ranks. This ensures that the uniform distribution of the rank vector, i.e., the result of Lemma 1, still holds, but we only obtain

$$S^{\text{test}}_{(\eta)} \le S_{(i)} \Longleftarrow R_\eta - \eta + 1 \le i.$$

instead of relation (7). Nevertheless, this still leads to inequality (8), provided that the second equality is replaced with an inequality.

6.3. *Proof of Theorem 1*

We need to show that for each k such that H_k is true, $x \mapsto \mathbb{P}\{(p_1, \ldots, p_K) \in A \mid p_k = x\}$ is nondecreasing over its domain of definition (i.e., over the set of xs where the probability is well-defined), for any increasing set $A \subset \mathbb{R}^K$. Without loss of generality, suppose H_1 is true, and fix any increasing set $A \subset \mathbb{R}^K$ and $x \in (0, 1)$.

For conciseness, we denote the comparison sample size as $m := n_1$, and the comparison scores as $S_{n+j} := S^{(1)}_j$, $j \in [m]$. Let $\bar{S}_{(i)}, i \in [n+m]$ be the i-th order statistic of $S_1, \ldots, S_{n+m}$—we write $\bar{S}_{(i)}$ to distinguish it from the i-th order statistic $S_{(i)}$ of $S_1, \ldots, S_n$. Let $\bar{S}_\uparrow = (\bar{S}_{(1)}, \ldots, \bar{S}_{(n+m)})$ and $S_\uparrow = (S_{(1)}, \ldots, S_{(n)})$. Let us define $R_1, R_2, \ldots, R_m$ as in Lemma 1. For simplicity, write $\eta := \eta_1$.

By definition, the batch conformal p-value p_1 from (2) can be written as

$$p_1 = \sum_{i=1}^{n} w_i \cdot \mathbf{1}\{S^{(1)}_{(\eta)} \le S_{(i)}\} + w_{n+1} = \sum_{i: S_{(i)} \ge \bar{S}_{(R_\eta)}} w_i + w_{n+1} = \sum_{i \ge R_\eta - \eta + 1} w_i + w_{n+1},$$

where $w_i = \binom{i+\eta-2}{\eta-1}\binom{n+m-i-\eta+1}{m-\eta}/\binom{n+m}{m}$ for $i \in [n+1]$. Therefore, the event $p_1 = x$ can be written in the form $R_\eta = r$ for some $r = r(x) \in [n+m]$. Further, by inspection, p_1 is a decreasing function of R_η. Putting everything together, our task reduces to proving that $r \mapsto \mathbb{P}\left\{(p_1, \ldots, p_K) \in A \,\middle|\, R_\eta = r\right\}$ is non-increasing in r.

Recalling $S_\uparrow = (S_{(1)}, \ldots, S_{(n)})$, observe that

$$\mathbb{P}\left\{(p_1, \ldots, p_K) \in A \;\middle|\; R_\eta = r\right\} = \mathbb{E}\left[\mathbb{P}\left\{(p_1, \ldots, p_K) \in A \;\middle|\; R_\eta = r, S_\uparrow\right\} \;\middle|\; R_\eta = r\right]$$
$$= \mathbb{E}_{S_\uparrow \sim P_{S_\uparrow | R_\eta = r}}\left[\mathbb{P}\left\{(p_1, \ldots, p_K) \in A \;\middle|\; S_\uparrow\right\}\right]. \quad (9)$$

Now we examine the distribution of $S_\uparrow$ given $R_\eta = r$. By definition of $R_1, \ldots, R_m$ from Lemma 1 as the ordered ranks of $S_{n+1}, \ldots, S_{n+m}$ among $\bar{S}_\uparrow = (\bar{S}_{(1)}, \bar{S}_{(2)}, \ldots, \bar{S}_{(n+m)})$, we can write the remaining ordered ranks $S_\uparrow = (S_{(1)}, \ldots, S_{(n)})$ as $\bar{S}_\uparrow$ with the coordinates $\bar{S}_{(R_j)}, j \in [m]$, removed:

$$S_\uparrow = (\bar{S}_{(1)}, \ldots, \bar{S}_{(R_1-1)}, \bar{S}_{(R_1+1)}, \ldots, \bar{S}_{(R_2-1)}, \bar{S}_{(R_2+1)}, \ldots, \bar{S}_{(R_m-1)}, \bar{S}_{(R_m+1)}, \ldots, \bar{S}_{(n+m)}). \quad (10)$$

Thus, $S_\uparrow$ is fully determined by $\bar{S}_\uparrow$ and $(R_1, \ldots, R_m)$. Therefore, the conditional distribution of $S_\uparrow$ given $\bar{S}_\uparrow$ and $R_\eta = r$ is determined by the conditional distribution of $(R_1, \ldots, R_m)$ given $\bar{S}_\uparrow$ and $R_\eta = r$. We will leverage this result in the proof.

Next, we will show the following lemma, which allows to represent conditional distributions of $S_\uparrow \mid R_\eta$ for two different values of R_η as a jointly distributed pair of random vectors decreasing in R_η. Below, for two random objects U, V, $U \overset{d}{=} V$ denotes that they have the same distribution.

LEMMA 3 (ALMOST SURE REPRESENTATION OF CONDITIONAL DISTRIBUTION OF $S_\uparrow \mid R_\eta$). *For any $r_1, r_2 \in [n+m]$, $r_1 < r_2$, there exist random vectors V_1 and V_2 over $\mathbb{R}^n$ such that the following two conditions hold with $S_\uparrow = (S_{(1)}, \ldots, S_{(n)})$:*

(1) $V_1 \overset{d}{=} (S_\uparrow \mid R_\eta = r_1)$ *and* $V_2 \overset{d}{=} (S_\uparrow \mid R_\eta = r_2)$ (2) $V_1 \geq V_2$ almost surely.

Proof. We first draw $\bar{S}_\uparrow = (\bar{S}_{(1)}, \ldots, \bar{S}_{(n+m)})$, and then show that there exist random vectors $(\tilde{R}_1, \ldots, \tilde{R}_m)$ and $(\hat{R}_1, \ldots, \hat{R}_m)$ such that the following conditions hold:

$$\begin{aligned} &(\tilde{R}_1, \ldots, \tilde{R}_m) \mid \bar{S}_\uparrow \overset{d}{=} (R_1, \ldots, R_m) \mid R_\eta = r_1, \bar{S}_\uparrow \\ &(\hat{R}_1, \ldots, \hat{R}_m) \mid \bar{S}_\uparrow \overset{d}{=} (R_1, \ldots, R_m) \mid R_\eta = r_2, \bar{S}_\uparrow \\ &(\tilde{R}_1, \ldots, \tilde{R}_m) \leq (\hat{R}_1, \ldots, \hat{R}_m) \text{ almost surely.} \end{aligned} \quad (11)$$

Then we will argue that the claim of the lemma follows from the observation (10), and in particular since $S_\uparrow$ is determined by $\bar{S}_\uparrow$ and $(R_1, \ldots, R_m)$.

Now, we condition on the event $\bar{S}_\uparrow = \bar{s}_\uparrow$ for some realization $\bar{s}_\uparrow$, and construct $\tilde{R}_j$s and $\hat{R}_j$s by induction on m. First, we observe that for any $t \in [m]$, $t \geq 2$, and $q, q' \in [m+n]$, $q \leq q'$, there exist random variables $\tilde{R}_{t-1,q,q'}$ and $\hat{R}_{t-1,q,q'}$ such that the following conditions hold.

$$\begin{aligned} &\tilde{R}_{t-1,q,q'} \mid \bar{S}_\uparrow = \bar{s}_\uparrow \overset{d}{=} R_{t-1} \mid R_t = q, \bar{S}_\uparrow = \bar{s}_\uparrow, \\ &\hat{R}_{t-1,q,q'} \mid \bar{S}_\uparrow = \bar{s}_\uparrow \overset{d}{=} R_{t-1} \mid R_t = q', \bar{S}_\uparrow = \bar{s}_\uparrow, \\ &\tilde{R}_{t-1,q,q'} \leq \hat{R}_{t-1,q,q'} \quad \text{almost surely.} \end{aligned}$$

This is a direct consequence of the second claim of Lemma 2 and Strassen's theorem [Strassen, 1965], which implies that for any random variables X and Y such that X stochastically dominates Y, there exist random variables X' and Y' such that $X' \overset{d}{=} X$, $Y' \overset{d}{=} Y$, and $X' \geq Y'$ almost surely.

Similarly, for any t and $q \leq q'$, there exist random variables $\tilde{R}'_{t+1,q,q'}$ and $\hat{R}'_{t+1,q,q'}$ such that

$$\tilde{R}'_{t+1,q,q'} \mid \bar{S}_{\uparrow} = \bar{s}_{\uparrow} \overset{d}{=} R_{t+1} \mid R_t = q, \bar{S}_{\uparrow} = \bar{s}_{\uparrow},$$
$$\hat{R}'_{t+1,q,q'} \mid \bar{S}_{\uparrow} = \bar{s}_{\uparrow} \overset{d}{=} R_{t+1} \mid R_t = q', \bar{S}_{\uparrow} = \bar{s}_{\uparrow},$$
$$\tilde{R}'_{t+1,q,q'} \leq \hat{R}'_{t+1,q,q'} \quad \text{almost surely}$$

hold. Moreover, we can construct the rank pairs $(\tilde{R}_{t,q,q'}, \hat{R}_{t,q,q'})$s and $(\tilde{R}'_{t,q,q'}, \hat{R}'_{t,q,q'})$s to be jointly independent and also independent of the actual ranks $R_1, \ldots, R_m$ (conditional on $\bar{S}_{\uparrow} = \bar{s}_{\uparrow}$)—by applying Strassen's theorem separately for the two cases.

Next, define the $\tilde{R}_j$s and $\hat{R}_j$s as follows.

1. Set $\tilde{R}_\eta = r_1$ and $\hat{R}_\eta = r_2$.
2. For $j = \eta - 1, \ldots, 2, 1$, define $\tilde{R}_j = \tilde{R}_{j,\tilde{R}_{j+1},\hat{R}_{j+1}}$ and $\hat{R}_j = \hat{R}_{j,\tilde{R}_{j+1},\hat{R}_{j+1}}$.
3. For $j = \eta + 1, \eta + 2, \ldots, m$, define $\tilde{R}_j = \tilde{R}'_{j,\tilde{R}_{j-1},\hat{R}_{j-1}}$ and $\hat{R}_j = \hat{R}'_{j,\tilde{R}_{j-1},\hat{R}_{j-1}}$.

The above construction is well defined, since for each $j \in [m + n]$, $\tilde{R}_j \leq \hat{R}_j$ holds by induction. Then, observe that for each $j < \eta$,

$$\begin{aligned}
&\tilde{R}_j \mid \tilde{R}_{j+1} = q, \hat{R}_{j+1} = q', \tilde{R}_{j+2}, \tilde{R}_{j+3}, \ldots, \tilde{R}_\eta, \bar{S}_{\uparrow} = \bar{s}_{\uparrow} \\
&\overset{d}{=} \tilde{R}_{j,q,q'} \mid \tilde{R}_{j+1} = q, \hat{R}_{j+1} = q', \tilde{R}_{j+2}, \tilde{R}_{j+3}, \ldots, \tilde{R}_\eta, \bar{S}_{\uparrow} = \bar{s}_{\uparrow} \qquad \text{by definition of } \tilde{R}_j \\
&\overset{d}{=} \tilde{R}_{j,q,q'} \mid \bar{S}_{\uparrow} = \bar{s}_{\uparrow} \qquad \text{by the independence of the constructed rank pairs} \\
&\overset{d}{=} R_j \mid R_{j+1} = q, \bar{S}_{\uparrow} = \bar{s}_{\uparrow} \qquad \text{by the construction of } \tilde{R}_{j,q,q'}.
\end{aligned}$$

Since the above holds for any $q \in [m + n]$ and the final distribution does not depend on $q' \in [m + n]$, this implies

$$\begin{aligned}
(\tilde{R}_j \mid \tilde{R}_{j+1}, \tilde{R}_{j+2}, \ldots, \tilde{R}_\eta, \bar{S}_{\uparrow} = \bar{s}_{\uparrow}) &\overset{d}{=} R_j \mid R_{j+1}, \bar{S}_{\uparrow} = \bar{s}_{\uparrow} \\
&\overset{d}{=} R_j \mid R_{j+1}, R_{j+2}, \ldots, R_\eta, \bar{S}_{\uparrow} = \bar{s}_{\uparrow},
\end{aligned}$$

where the second equality follows by the first claim of Lemma 2. By an analogous argument, we have

$$(\hat{R}_j \mid \hat{R}_{j+1}, \hat{R}_{j+2}, \ldots, \hat{R}_\eta, \bar{S}_{\uparrow} = \bar{s}_{\uparrow}) \overset{d}{=} R_j \mid R_{j+1}, R_{j+2}, \ldots, R_\eta, \bar{S}_{\uparrow} = \bar{s}_{\uparrow}$$

for any $j < \eta$. Next, for each $j > \eta$, we have by similar arguments that

$$(\tilde{R}_j \mid \tilde{R}_{j-1}, \tilde{R}_{j-2}, \ldots, \tilde{R}_\eta, \ldots, \tilde{R}_1, \bar{S}_{\uparrow} = \bar{s}_{\uparrow}) \overset{d}{=} R_j \mid R_{j-1}, R_{j-2}, \ldots, R_\eta, \ldots, R_1, \bar{S}_{\uparrow} = \bar{s}_{\uparrow},$$
$$(\hat{R}_j \mid \hat{R}_{j-1}, \hat{R}_{j-2}, \ldots, \hat{R}_\eta, \ldots, \hat{R}_1, \bar{S}_{\uparrow} = \bar{s}_{\uparrow}) \overset{d}{=} R_j \mid R_{j-1}, R_{j-2}, \ldots, R_\eta, \ldots, R_1, \bar{S}_{\uparrow} = \bar{s}_{\uparrow}.$$

Therefore, putting everything together, the random vectors $(\tilde{R}_1, \ldots, \tilde{R}_m)$ and $(\hat{R}_1, \ldots, \hat{R}_m)$ satisfy the conditions in (11). Now we set V_1 and V_2 as

$$V_1 = (\bar{S}_{(1)}, \ldots, \bar{S}_{(\tilde{R}_1-1)}, \bar{S}_{(\tilde{R}_1+1)}, \ldots, \bar{S}_{(\tilde{R}_2-1)}, \bar{S}_{(\tilde{R}_2+1)}, \ldots, \bar{S}_{(\tilde{R}_m-1)}, \bar{S}_{(\tilde{R}_m+1)}, \ldots, \bar{S}_{(n+m)}),$$
$$V_2 = (\bar{S}_{(1)}, \ldots, \bar{S}_{(\hat{R}_1-1)}, \bar{S}_{(\hat{R}_1+1)}, \ldots, \bar{S}_{(\hat{R}_2-1)}, \bar{S}_{(\hat{R}_2+1)}, \ldots, \bar{S}_{(\hat{R}_m-1)}, \bar{S}_{(\hat{R}_m+1)}, \ldots, \bar{S}_{(n+m)}).$$

Then $V_1 \succeq V_2$ holds almost surely, since $(\tilde{R}_1, \ldots, \tilde{R}_m) \preceq (\hat{R}_1, \ldots, \hat{R}_m)$ holds with probability one.

Furthermore, from (11), we have $V_1 \mid \bar{S}_{\uparrow} \stackrel{d}{=} (S_{\uparrow} \mid R_{\eta} = r_1, \bar{S}_{\uparrow})$ and $V_2 \mid \bar{S}_{\uparrow} \stackrel{d}{=} (S_{\uparrow} \mid R_{\eta} = r_2, \bar{S}_{\uparrow})$. By marginalizing over $\bar{S}_{\uparrow}$, we also obtain $V_1 \stackrel{d}{=} S_{\uparrow} \mid R_{\eta} = r_1)$ and $V_2 \stackrel{d}{=} (S_{\uparrow} \mid R_{\eta} = r_2)$. $\square$

Note also that from the between-group independence assumption, we have

$$(V_1, S^{(2)}_{(\eta_2)}, \dots, S^{(K)}_{(\eta_K)}) \stackrel{d}{=} ((S_{\uparrow}, S^{(2)}_{(\eta_2)}, \dots, S^{(K)}_{(\eta_K)}) \mid R_{\eta} = r_1), \text{ and}$$
$$(V_2, S^{(2)}_{(\eta_2)}, \dots, S^{(K)}_{(\eta_K)}) \stackrel{d}{=} ((S_{\uparrow}, S^{(2)}_{(\eta_2)}, \dots, S^{(K)}_{(\eta_K)}) \mid R_{\eta} = r_2).$$

Now, for each $k \in [K]$, let $p_k^{(1)}$ and $p_k^{(2)}$ be defined as in (2), with $S_{\uparrow}$ replaced by V_1 and V_2, respectively. Now, $p_j^{(1)} \geq p_j^{(2)}$ holds almost surely for all $j \in [K]$ by the definition (2) and the fact that $V_1 \geq V_2$ holds almost surely. Since A is an increasing set, from (9) and the above results, we thus have that

$$\begin{aligned}\mathbb{P}\left\{(p_1, \dots, p_K) \in A \,\middle|\, R_{\eta} = r_1\right\} &= \mathbb{E}_{S_{\uparrow} \sim P_{S_{\uparrow} \mid R_{\eta} = r}}\left[\mathbb{P}\left\{(p_1, \dots, p_K) \in A \,\middle|\, S_{\uparrow}\right\}\right] \\ &= \mathbb{E}\left[\mathbb{P}\left\{(p_1^{(1)}, \dots, p_k^{(1)}) \in A \,\middle|\, V_1\right\}\right] = \mathbb{P}\left\{(p_1^{(1)}, \dots, p_k^{(1)}) \in A\right\} \\ &\geq \mathbb{P}\left\{(p_1^{(2)}, \dots, p_K^{(2)}) \in A\right\} = \mathbb{P}\left\{(p_1, \dots, p_K) \in A \,\middle|\, R_{\eta} = r_2\right\},\end{aligned}$$

as desired.

The second claim of Theorem 1 follows from the result of Benjamini and Yekutieli [2001, Theorem 1.2].

6.4. *Proof of Theorem 1*

Let us define $R_1 < \ldots < R_m$ as in the proof of the validity of batch conformal p-values. By the observation in (7), we have the following equivalence:

$$\begin{aligned} S^{\text{test}}_{(\eta_1)} \leq S_{(\tilde{\eta}_1 + t)}, S^{\text{test}}_{(\eta_2)} \leq S_{(\tilde{\eta}_2 + t)} &\iff R_{\eta_1} - \eta_1 + 1 \leq \tilde{\eta}_1 + t \text{ and } R_{\eta_2} - \eta_2 + 1 \leq \tilde{\eta}_2 + t \\ &\iff T \leq t, \text{ where } T = \max\{R_{\eta_1} - \eta_1 - \tilde{\eta}_1 + 1, R_{\eta_2} - \eta_2 - \tilde{\eta}_2 + 1\}. \end{aligned}$$

Next, we compute the probability mass function of T. For $-\tilde{\eta}_1 + 1 \leq t \leq n - \tilde{\eta}_2 + 1$,

$$\begin{aligned} \mathbb{P}\{T = t\} &= \mathbb{P}\left\{\max\{R_{\eta_1} - \eta_1 - \tilde{\eta}_1 + 1, R_{\eta_2} - \eta_2 - \tilde{\eta}_2 + 1\} = t\right\} \\ &= \sum_{\substack{t_1, t_2 \,:\, \max\{t_1, t_2\} = t \\ 1 \leq \eta_1 + \tilde{\eta}_1 + t_1 + 1 < \eta_2 + \tilde{\eta}_2 + t_2 + 1 \leq n + m}} \mathbb{P}\left\{R_{\eta_1} - \eta_1 - \tilde{\eta}_1 + 1 = t_1, R_{\eta_2} - \eta_2 - \tilde{\eta}_2 + 1 = t_2\right\} \\ &= \sum_{\substack{t_1, t_2 \,:\, \max\{t_1, t_2\} = t \\ 1 \leq \eta_1 + \tilde{\eta}_1 + t_1 + 1 < \eta_2 + \tilde{\eta}_2 + t_2 + 1 \leq n + m}} \mathbb{P}\left\{R_{\eta_1} = t_1 + \eta_1 + \tilde{\eta}_1 - 1, R_{\eta_2} = t_2 + \eta_2 + \tilde{\eta}_2 - 1\right\} \\ &= \sum_{\substack{t_1, t_2 \,:\, \max\{t_1, t_2\} = t \\ 1 \leq \eta_1 + \tilde{\eta}_1 + t_1 + 1 < \eta_2 + \tilde{\eta}_2 + t_2 + 1 \leq n + m}} \frac{\binom{\eta_1 + \tilde{\eta}_1 + t_1 - 2}{\eta_1 - 1}\binom{\eta_2 + \tilde{\eta}_2 + t_2 - \eta_1 + \tilde{\eta}_1 + t_1 - 1}{\eta_2 - \eta_1 - 1}\binom{n + m - \eta_2 - \tilde{\eta}_2 - t_2 + 1}{m - \eta_2}}{\binom{n + m}{m}} = w_t. \end{aligned}$$

In the last line, we have used the definition of w_t from (3). Therefore, the cumulative distribution function of T is given by $\tau \mapsto F_T(\tau) = \sum_{t=-\tilde{\eta}_1+1}^{n-\tilde{\eta}_2+1} w_t \mathbb{1}\{t \le \tau\}$, and thus we have that

$$\mathbb{P}\{p \le \alpha\} = \mathbb{P}\left\{\sum_{t=-\tilde{\eta}_1+1}^{n-\tilde{\eta}_2+1} w_t \cdot \mathbb{1}\left\{S^{\text{test}}_{(\eta_1)} \le S_{(\tilde{\eta}_1+t)}, S^{\text{test}}_{(\eta_2)} \le S_{(\tilde{\eta}_2+t)}\right\} \le \alpha\right\}$$
$$= \mathbb{P}\left\{\sum_{t=-\tilde{\eta}_1+1}^{n-\tilde{\eta}_2+1} w_t \mathbb{1}\{T \le t\} \le \alpha\right\} = \mathbb{P}\{F_T(T) \le \alpha\} \le \alpha$$

holds for any $\alpha \in (0,1)$. This finishes the proof.

6.5. *Proof of Proposition 1*

By the definition of r_α, the event $\{p \le \alpha\}$ is equivalent to the event $\{R \ge r_\alpha\}$. Conditional on $T_{(\eta)} = x$, the indicators $\mathbb{1}\{S_i < x\}$, $i \in [n]$, are i.i.d. Bernoulli random variables with success probability $F_0(x)$, because the S_i are i.i.d. with cdf F_0 and are independent of $T_{(\eta)}$. Therefore $R - 1 \mid T_{(\eta)} = x \sim \text{Bin}\big(n, F_0(x)\big)$. It follows that

$$\mathbb{P}_{F_0,F_1}\left\{p \le \alpha \,\middle|\, T_{(\eta)} = x\right\} = \mathbb{P}\left\{R \ge r_\alpha \,\middle|\, T_{(\eta)} = x\right\} = \sum_{j=r_\alpha-1}^{n} \binom{n}{j} F_0(x)^j \big(1 - F_0(x)\big)^{n-j}.$$

Marginalizing over $T_{(\eta)}$ yields the first statement.

Next, recall that if $U \sim \text{Beta}(\eta, m-\eta+1)$, then $T_{(\eta)} \overset{d}{=} F_1^{-1}(U)$. Substituting $x = F_1^{-1}(U)$ in the previous expression proves the second claim.

6.6. *Proof of Corollary 1*

The proof applies the following two well known results: First, by the probability integral transform, if X has continuous cdf F, then $F(X) \sim \text{Unif}([0,1])$ [Rosenblatt, 1952]. Second, the η-th order statistic of m i.i.d. Unif(0, 1) variables has the $\text{Beta}(\eta, m-\eta+1)$ distribution.

Under the null, the transformed scores $F_0(T_1), \dots, F_0(T_m)$ are i.i.d. $\text{Unif}([0,1])$ by the probability integral transform. Hence $F_0(T_{(\eta)}) \sim \text{Beta}(\eta, m-\eta+1)$ by the order-statistic law previously used. It follows that $B_{\eta,m-\eta+1}(F_0(T_{(\eta)})) \sim \text{Unif}([0,1])$, and thus $p_\infty = 1 - B_{\eta,m-\eta+1}(F_0(T_{(\eta)})) \sim \text{Unif}([0,1])$ as well.

Now recall our assumption that $F_1(x) = F_0(x-\mu)$. If $U \sim \text{Beta}(\eta, m-\eta+1)$, then by a similar argument we have $T_{(\eta)} \overset{d}{=} F_1^{-1}(U)$. Since $F_1^{-1}(u) = F_0^{-1}(u) + \mu$, this gives $F_0(T_{(\eta)}) \overset{d}{=} F_0(F_0^{-1}(U) + \mu)$. Next, observe that

$$p_\infty \le t \iff 1 - B_{\eta,m-\eta+1}(F_0(T_{(\eta)})) \le t \iff B_{\eta,m-\eta+1}(F_0(T_{(\eta)})) \ge 1 - t \iff F_0(T_{(\eta)}) \ge b_t,$$

since $B_{\eta,m-\eta+1}$ is continuous and strictly increasing on $[0,1]$. Consequently,

$$H_{\mu,\eta}(t) = \mathbb{P}\{p_\infty \le t\} = \mathbb{P}\left\{F_0(F_0^{-1}(U) + \mu) \ge b_t\right\}.$$

Since F_0 is nondecreasing, the event inside the probability is equivalent to $F_0^{-1}(U) + \mu \ge F_0^{-1}(b_t)$, which is again equivalent to $U \ge F_0(F_0^{-1}(b_t) - \mu)$. Therefore

$$H_{\mu,\eta}(t) = \mathbb{P}\left\{U \ge F_0(F_0^{-1}(b_t) - \mu)\right\} = 1 - B_{\eta,m-\eta+1}\big(F_0(F_0^{-1}(b_t) - \mu)\big).$$

Finally, $\mu \mapsto F_0(F_0^{-1}(b_t) - \mu)$ is nonincreasing and $B_{\eta,m-\eta+1}$ is nondecreasing, implying that $H_{\mu,\eta}(t)$ is nondecreasing in μ.

6.7. *Proof of Corollary 2*

We first introduce the following results.

LEMMA 4 (EXACT THRESHOLD REPRESENTATION OF THE BENJAMINI–HOCHBERG PROCEDURE). *Let $p_1, \dots, p_K \in [0,1]$, let $p_{(1)} \le \cdots \le p_{(K)}$ be their order statistics, and define $\hat{k}_K = \max\{k \in [K] : p_{(k)} \le \alpha k/K\}$, with the convention $\hat{k}_K = 0$ if the set is empty. Define $\hat{\tau}_K = \alpha \hat{k}_K/K$ and $\hat{G}_K(t) = K^{-1}\sum_{i=1}^K \mathbb{1}\{p_i \le t\}$. Then $\hat{\tau}_K = \sup\{t \in [0,1] : \hat{G}_K(t) \ge t/\alpha\}$, and the Benjamini–Hochberg rejection set is equal to $\{i \in [K] : p_i \le \hat{\tau}_K\}$.*

Lemma 4 follows from the standard exact threshold representation—see e.g., the representations in Genovese and Wasserman [2002], Genovese and Wasserman [2004, Section 2.3], and Neuvial [2013, equation (6)].

The next result follows from the classical asymptotic theory under independent mixture models [Genovese and Wasserman, 2002, 2004, Chi, 2007, Neuvial, 2008, 2013, Ferreira and Zwinderman, 2006a]. Here we include a self-contained proof that enables us to plug in the explicit form of $H_{\mu,\eta}$ from Corollary 1.

PROPOSITION 2 (ASYMPTOTIC BENJAMINI–HOCHBERG THRESHOLD AND POWER). *Fix $\alpha \in (0,1)$ and $\pi_0 \in (0,1)$, and write $\pi_1 = 1-\pi_0$. For each $K \ge 1$, let $K_0 = \lfloor \pi_0 K \rfloor$ and $K_1 = K - K_0$. Consider independent p-values $(P_{1,K}, \dots, P_{K,K}) = (U_1, \dots, U_{K_0}, W_1, \dots, W_{K_1})$, where $U_1, \dots, U_{K_0} \overset{\text{i.i.d.}}{\sim} \mathrm{Unif}(0,1)$ and $W_1, \dots, W_{K_1} \overset{\text{i.i.d.}}{\sim} H_{\mu,\eta}$ from Corollary 1, independently of the U_i's. Let $\hat{G}_K(t) = K^{-1}\sum_{i=1}^K \mathbb{1}\left\{P_{i,K} \le t\right\}$ and let $G_{\mu,\eta}(t) = \pi_0 t + \pi_1 H_{\mu,\eta}(t)$. Let $\hat{\tau}_K$ be the Benjamini–Hochberg threshold at level α, as in Lemma 4. Define $\Psi(t) = G_{\mu,\eta}(t) - t/\alpha$ and $t_\star = \sup\{t \in [0,1] : \Psi(t) \ge 0\}$. Suppose that $t_\star \in (0,1)$ and that there exists $\delta_0 > 0$ such that $\Psi(t) > 0$ for every $t \in (t_\star - \delta_0, t_\star)$ and $\Psi(t) < 0$ for every $t \in (t_\star, t_\star + \delta_0)$.*

Then $t_\star$ is the largest solution of $G_{\mu,\eta}(t) = t/\alpha$, equivalently the largest solution of $\pi_0 t + \pi_1 H_{\mu,\eta}(t) = t/\alpha$. Moreover, $\hat{\tau}_K$ converges to $t_\star$ almost surely. For the empirical power $\widehat{\mathrm{Pow}}_K = K_1^{-1}\sum_{j=1}^{K_1} \mathbb{1}\left\{W_j \le \hat{\tau}_K\right\}$, it holds that $\widehat{\mathrm{Pow}}_K \to H_{\mu,\eta}(t_\star)$ almost surely and $\mathbb{E}[\widehat{\mathrm{Pow}}_K] \to H_{\mu,\eta}(t_\star)$. Finally, for each fixed non-null index j, $\mathbb{P}\left\{W_j \le \hat{\tau}_K\right\} \to H_{\mu,\eta}(t_\star)$.

Proof. Let $\hat{F}_{0,K}(t) = K_0^{-1}\sum_{i=1}^{K_0} \mathbb{1}\{U_i \le t\}$ and $\hat{F}_{1,K}(t) = K_1^{-1}\sum_{j=1}^{K_1} \mathbb{1}\left\{W_j \le t\right\}$. Then $\hat{G}_K(t) = \frac{K_0}{K}\hat{F}_{0,K}(t) + \frac{K_1}{K}\hat{F}_{1,K}(t)$ for every $t \in [0,1]$.

By Glivenko–Cantelli theorem [Glivenko, 1933, Cantelli, 1933], we have $\sup_{t\in[0,1]} |\hat{F}_{0,K}(t) - t| \to 0$ almost surely and $\sup_{t\in[0,1]} |\hat{F}_{1,K}(t) - H_{\mu,\eta}(t)| \to 0$ almost surely. Since $K_0/K \to \pi_0$ and $K_1/K \to \pi_1$, from the inequality

$$\sup_{t\in[0,1]} |\hat{G}_K(t) - G_{\mu,\eta}(t)| \le \frac{K_0}{K}\sup_{t\in[0,1]} |\hat{F}_{0,K}(t) - t| + \frac{K_1}{K}\sup_{t\in[0,1]} |\hat{F}_{1,K}(t) - H_{\mu,\eta}(t)| + \left|\frac{K_0}{K} - \pi_0\right| + \left|\frac{K_1}{K} - \pi_1\right|,$$

it follows that $\sup_{t\in[0,1]} |\hat{G}_K(t) - G_{\mu,\eta}(t)| \to 0$ almost surely. Equivalently, for $\hat{\Psi}_K(t)$ defined as $\hat{\Psi}_K(t) = \hat{G}_K(t) - t/\alpha$, it holds that $\sup_{t\in[0,1]} |\hat{\Psi}_K(t) - \Psi(t)| \to 0$ almost surely. This uniform empirical-cdf approximation follows the logic of asymptotic Benjamini–Hochberg analyses of Genovese and Wasserman [2002], Chi [2007], Neuvial [2008, 2013].

Since $H_{\mu,\eta}$ is continuous by Corollary 1, the functions $G_{\mu,\eta}$ and Ψ are also continuous. By assumption, $\Psi(t) > 0$ for all $t \in (t_\star - \delta_0, t_\star)$ and $\Psi(t) < 0$ for all $t \in (t_\star, t_\star + \delta_0)$, and the continuity of the function implies $\Psi(t_\star) = 0$. Therefore, we have $G_{\mu,\eta}(t_\star) = t_\star/\alpha$. For any

other solution t of $G_{\mu,\eta}(t) = t/\alpha$, we have $\Psi(t) = 0$ and thus t belongs to the set $\{u \in [0,1] : \Psi(u) \geq 0\}$ whose supremum is $t_\star$, implying $t \leq t_\star$. Hence $t_\star$ is the largest solution of $G_{\mu,\eta}(t) = \pi_0 t + \pi_1 H_{\mu,\eta}(t) = t/\alpha$.

We now prove that $\hat{\tau}_K \longrightarrow t_\star$ almost surely. Recall that the event $\sup_{t\in[0,1]} |\hat{\Psi}_K(t) - \Psi(t)| \longrightarrow 0$ holds with probability one, and assume this event holds from now on. Let $\varepsilon \in (0, \min\{t_\star, 1 - t_\star\})$. Since $\Psi(t) < 0$ for every $t \in [t_\star + \varepsilon, 1]$, we have from the continuity that the maximum of Ψ on the compact interval $[t_\star + \varepsilon, 1]$ is strictly negative. Define $c_+(\varepsilon) = -\max_{t\in[t_\star+\varepsilon,1]} \Psi(t) > 0$. For all sufficiently large K, we have $\sup_{t\in[0,1]} |\hat{\Psi}_K(t) - \Psi(t)| < c_+(\varepsilon)$. Therefore, for every $t \in [t_\star + \varepsilon, 1]$,

$$\hat{\Psi}_K(t) \leq \Psi(t) + |\hat{\Psi}_K(t) - \Psi(t)| < -c_+(\varepsilon) + c_+(\varepsilon) = 0.$$

Hence no $t \in [t_\star + \varepsilon, 1]$ belongs to $\{u : \hat{\Psi}_K(u) \geq 0\}$, and Lemma 4 implies that $\hat{\tau}_K < t_\star + \varepsilon$ for all sufficiently large K.

For the lower bound, let $\varepsilon_0 = \min\{\varepsilon, \delta_0\}$ and define $s_\varepsilon = t_\star - \varepsilon_0/2$. Then we have $s_\varepsilon \in (t_\star - \varepsilon, t_\star)$, and from the assumption we have $\Psi(s_\varepsilon) > 0$. Let $c_-(\varepsilon) = \Psi(s_\varepsilon) > 0$. For all sufficiently large K, we have $\sup_{t\in[0,1]} |\hat{\Psi}_K(t) - \Psi(t)| < c_-(\varepsilon)$. Therefore,

$$\hat{\Psi}_K(s_\varepsilon) \geq \Psi(s_\varepsilon) - |\hat{\Psi}_K(s_\varepsilon) - \Psi(s_\varepsilon)| > c_-(\varepsilon) - c_-(\varepsilon) = 0.$$

Hence s_ε belongs to the set $\{u : \hat{\Psi}_K(u) \geq 0\}$, and Lemma 4 implies $\hat{\tau}_K \geq s_\varepsilon > t_\star - \varepsilon$ for all sufficiently large K. Since this holds for an arbitraty ε, we have that $\hat{\tau}_K \longrightarrow t_\star$ almost surely.

Next, note that $\widehat{\mathrm{Pow}}_K = \hat{F}_{1,K}(\hat{\tau}_K)$ by definition. Therefore,

$$\left|\widehat{\mathrm{Pow}}_K - H_{\mu,\eta}(t_\star)\right| \leq \sup_{t\in[0,1]} |\hat{F}_{1,K}(t) - H_{\mu,\eta}(t)| + \left|H_{\mu,\eta}(\hat{\tau}_K) - H_{\mu,\eta}(t_\star)\right|.$$

The first term tends to 0 almost surely by the Glivenko–Cantelli theorem [Glivenko, 1933, Cantelli, 1933], and the second term tends to 0 almost surely since $\hat{\tau}_K \longrightarrow t_\star$ almost surely and $H_{\mu,\eta}$ is continuous. It follows that $\widehat{\mathrm{Pow}}_K \longrightarrow H_{\mu,\eta}(t_\star)$ almost surely. Since $0 \leq \widehat{\mathrm{Pow}}_K \leq 1$, the bounded convergence theorem yields $\mathbb{E}[\widehat{\mathrm{Pow}}_K] \longrightarrow H_{\mu,\eta}(t_\star)$.

Finally, since $W_1, \ldots, W_{K_1}$ are exchangeable and $\hat{\tau}_K$ is a symmetric measurable function of all the input p-values, the indicators $\mathbb{1}\left\{W_j \leq \hat{\tau}_K\right\}$ have the same expectation for all $j \in [K_1]$. Therefore,

$$\mathbb{E}[\widehat{\mathrm{Pow}}_K] = \mathbb{E}\left[\frac{1}{K_1}\sum_{j=1}^{K_1} \mathbb{1}\left\{W_j \leq \hat{\tau}_K\right\}\right] = \frac{1}{K_1}\sum_{j=1}^{K_1} \mathbb{E}\big[\mathbb{1}\left\{W_j \leq \hat{\tau}_K\right\}\big] = \mathbb{P}\left\{W_1 \leq \hat{\tau}_K\right\}.$$

This completes the proof. □

Corollary 2 follows from Corollary 1 and Proposition 2.

7. Positive regression dependence of one-sided two sample normal p-values

Here, we show that the oracle one-sided two sample p-values $(p_k)_{1\leq k\leq K}$, as defined in (12), satisfy positive regression dependence. Consequently, applying the Benjamini-Hochberg procedure to these p-values ensures valid false discovery rate control. The positive regression dependence property follows from Loper et al. [2022], but for clarity, we also provide here a formal statement and its proof.

PROPOSITION 3. *Suppose the null distribution is $\mathcal{N}(0,\sigma^2)$. Then the p-values $p_1, p_2, \ldots, p_K$ defined as*

$$p_k = \Phi\left(\frac{\bar{X}_{\mathrm{ref}} - \bar{X}_k}{\sqrt{\frac{\sigma^2}{n} + \frac{\sigma^2}{n_k}}}\right), \quad k = 1, 2, \ldots, K, \tag{12}$$

are positive regression dependent on the set of nulls.

7.1. *Proof of Proposition 3*

Fix an increasing set $A \subset \mathbb{R}^K$. We need to show that for each k such that H_k is true, as a function of $x \in (0,1)$, $x \mapsto \mathbb{P}\{(p_1, \ldots, p_K) \in A \mid p_k = x\}$ is nondecreasing. Without loss of generality, we assume that H_1 is true, and then prove the monotonicity of the above quantity for $k = 1$. Let $m := n_1$ for simplicity.

We first investigate the conditional distribution of $\bar{X}_{\mathrm{ref}}$ given $p_1 = x$. By the definition of p_1, the event $p_1 = x$ is equivalent to $\bar{X}_{\mathrm{ref}} - \bar{X}_1 = z$, where $z = \Phi^{-1}(x) \cdot \sigma\sqrt{\frac{1}{n} + \frac{1}{m}}$. Since the reference and the comparison datapoints are i.i.d. $\mathcal{N}(0,\sigma^2)$ under the null, the conditional distribution of $\bar{X}_{\mathrm{ref}}$ given $\bar{X}_{\mathrm{ref}} - \bar{X}_1 = z$ is $\mathcal{N}\left(\frac{m}{n+m}z, \frac{\sigma^2}{n+m}\right)$. Now take any $x' \in (0,1)$, $x' > x$ and let $z' = \Phi^{-1}(x') \cdot \sigma\sqrt{\frac{1}{n} + \frac{1}{m}}$. Since Φ^{-1} is an increasing function, we have $z' > z$. This implies that the distribution $\mathcal{N}\left(\frac{m}{n+m}z', \frac{\sigma^2}{n+m}\right)$ is stochastically larger than the distribution $\mathcal{N}\left(\frac{m}{n+m}z, \frac{\sigma^2}{n+m}\right)$. Therefore, putting everything together, there exist random variables V_1 and V_2 such that the following conditions hold.

$$(1)\ V \stackrel{d}{=} \bar{X}_{\mathrm{ref}} \mid p_1 = x, \quad (2)\ V' \stackrel{d}{=} \bar{X}_{\mathrm{ref}} \mid p_1 = x' \quad (3)\ V \le V' \text{ almost surely.}$$

Next, for each $k = 2, 3, \ldots, K$, define p_k^V as $p_k^V = \Phi\left(\frac{V - \bar{X}_k}{\sqrt{\frac{\sigma^2}{n} + \frac{\sigma^2}{n_k}}}\right)$, and define $p_k^{V'}$ similarly. By condition (3) above, $p_k^V \le p_k^{V'}$ holds almost surely, for each $k = 2, 3, \ldots, K$. Therefore, we have

$$\begin{aligned}\mathbb{P}\{(p_1, \ldots, p_K) \in A \mid p_k = x\} = \mathbb{P}\left\{(p_1^V, \ldots, p_K^V) \in A\right\} &\le \mathbb{P}\left\{(p_1^{V'}, \ldots, p_K^{V'}) \in A\right\} \\ &= \mathbb{P}\{(p_1, \ldots, p_K) \in A \mid p_k = x'\},\end{aligned}$$

where the first and the second equality apply conditions (1) and (2), respectively. This proves positive regression dependence.

8. EXPERIMENTS WITH CPS WORK HOURS DATA

In this section, we provide experimental results using the Current Population Survey (CPS) dataset.[2][3] This experiment is an example of a more straightforward illustration—without additional steps required to apply the procedure, as was necessary in the HALT-C dataset, where we had to account for unobserved counterfactual variables.

[2]This dataset was accessed at `https://murraylax.org/datasets/cpshours.csv`. The dataset has been analyzed at `https://murraylax.org/rtutorials/oneway-anova.html`. It includes data from more than 52,000 individuals over the age of 25 years that participated in the 2016 Current Population Survey.

[3]Code to reproduce the experiments in this section is available at `https://github.com/yhoon31/batch_conformal`.

The dataset consists of demographic information and education levels of various individuals, and the outcome of interest is their work hours. We form groups based on age, sex, race, and education level. Sex has two categories: "Male" and "Female". Race has five categories: "White", "Black", "Asian/Pacific Islander", "American Indian/Aleut/Eskimo", and "Other". Education level has four categories: "High school", "Some college", "Four-year degree", and "Advanced degree". Age is divided into four groups: "25–39", "40–49", "50–59", and "60+". Therefore, there are $2 \cdot 5 \cdot 4 \cdot 4 = 160$ possible groups, and we include 157 groups in the experiments, each of which has at least five datapoints.

The group with the largest sample size—Female/White/Some college/25–39—is set as the reference group. Although the original group contains 2,912 datapoints, we randomly select 100 to simulate a small-sample setting, where distinguishing between distributions is more difficult, and use these as the reference data. Similarly, for comparison groups with more than 50 datapoints, we randomly select 50 to use as the comparison data.

We run our procedure using the outcome variable—work hours—itself as the score, with three test statistics: the median and the quartiles, as in the experiment with the HALT-C dataset. This leads to the detection of groups whose overall work hours are significantly higher compared to the reference group. Table 1 shows the list of selected groups from the procedure at levels $\alpha = 0.01$ and 0.05, demonstrating that there are larger differences in higher quantiles.

References

Yoav Benjamini and Daniel Yekutieli. The control of the false discovery rate in multiple testing under dependency. *Annals of statistics*, pages 1165–1188, 2001.

Munmun Biswas, Minerva Mukhopadhyay, and Anil K. Ghosh. A distribution-free two-sample run test applicable to high-dimensional data. *Biometrika*, 101(4):913–926, 10 2014.

F. P. Cantelli. Sulla determinazione empirica delle leggi di probabilità. *Giornale dell'Istituto Italiano degli Attuari*, 4:421–424, 1933.

Zhiyi Chi. On the performance of FDR control: Constraints and a partial solution. *The Annals of Statistics*, 35(4): 1409–1431, 2007. .

Meyer Dwass. Modified randomization tests for nonparametric hypotheses. *The Annals of Mathematical Statistics*, pages 181–187, 1957.

T Eden and F Yates. On the validity of fisher's z test when applied to an actual example of non-normal data. *The Journal of Agricultural Science*, 23(1):6–17, 1933.

Bradley Efron. Large-scale simultaneous hypothesis testing: The choice of a null hypothesis. *Journal of the American Statistical Association*, 99(465):96–104, 2004. .

Bradley Efron. Size, power and false discovery rates. *The Annals of Statistics*, 35(4):1351–1377, 2007. .

Bradley Efron. Microarrays, empirical bayes and the two-groups model. *Statistical Science*, 23(1):1–22, 2008. .

J. A. Ferreira and A. H. Zwinderman. On the benjamini–hochberg method. *The Annals of Statistics*, 34(4):1827–1849, 2006a. .

José António Ferreira and Aeilko H. Zwinderman. Approximate power and sample size calculations with the benjamini–hochberg method. *The International Journal of Biostatistics*, 2(1):Article 8, 2006b. .

Ronald Aylmer Fisher. *The design of experiments*. Oliver and Boyd, 1935.

Christopher Genovese and Larry Wasserman. Operating characteristics and extensions of the false discovery rate procedure. *Journal of the Royal Statistical Society: Series B (Statistical Methodology)*, 64(3):499–517, 2002. .

Christopher Genovese and Larry Wasserman. A stochastic process approach to false discovery control. *The Annals of Statistics*, 32(3):1035–1061, 2004. .

V. Glivenko. Sulla determinazione empirica delle leggi di probabilità. *Giornale dell'Istituto Italiano degli Attuari*, 4: 92–99, 1933.

Deborah H. Glueck, Jan Mandel, Anis Karimpour-Fard, Lawrence Hunter, and Keith E. Muller. Exact calculations of average power for the benjamini–hochberg procedure. *The International Journal of Biostatistics*, 4(1):Article 11, 2008. .

Arthur Gretton, Karsten M Borgwardt, Malte J Rasch, Bernhard Schölkopf, and Alexander Smola. A kernel two-sample test. *The Journal of Machine Learning Research*, 13(1):723–773, 2012.

Grant Izmirlian. Strong consistency and asymptotic normality for quantities related to the benjamini–hochberg false discovery rate procedure. *Statistics & Probability Letters*, 160:108713, 2020. .

				Q_1		Q_2		Q_3	
Sex	Race	Education	Age	0.01	0.05	0.01	0.05	0.01	0.05
M	American Indian/Aleut/Eskimo	High School	25–39	–	–	–	✓	–	–
M	Other	High School	50–59	–	✓	–	–	–	–
M	White	High School	25–39	–	–	–	✓	–	–
M	White	High School	40–49	–	–	–	✓	–	✓
M	American Indian/Aleut/Eskimo	Some College	25–39	–	–	–	✓	–	–
F	Black	Some College	40–49	–	–	–	✓	–	–
M	Black	Some College	25–39	–	–	–	–	–	✓
M	Black	Some College	40–49	–	–	–	–	–	✓
M	Black	Some College	50–59	–	–	–	✓	–	–
M	Other	Some College	25–39	–	–	–	✓	–	✓
M	Other	Some College	40–49	–	✓	✓	✓	✓	✓
M	White	Some College	25–39	–	✓	✓	✓	–	–
M	White	Some College	40–49	–	✓	✓	✓	✓	✓
M	White	Some College	50–59	–	–	–	–	–	✓
M	White	Some College	60+	–	–	–	–	✓	✓
M	Asian/Pacific Islander	Four Year	25–39	–	–	–	✓	–	–
M	Asian/Pacific Islander	Four Year	40–49	–	✓	✓	✓	–	✓
M	Asian/Pacific Islander	Four Year	50–59	–	✓	–	–	–	–
M	Asian/Pacific Islander	Four Year	60+	–	✓	–	–	–	–
M	Black	Four Year	40–49	–	–	–	✓	–	✓
M	Black	Four Year	50–59	–	–	–	–	–	✓
M	Other	Four Year	50–59	–	✓	–	✓	–	–
F	White	Four Year	40–49	–	–	–	✓	–	✓
M	White	Four Year	25–39	–	–	–	✓	–	✓
M	White	Four Year	40–49	–	✓	✓	✓	✓	✓
M	White	Four Year	50–59	–	✓	✓	✓	–	✓
M	White	Four Year	60+	–	–	–	–	–	✓
F	Asian/Pacific Islander	Advanced	40–49	–	–	–	✓	–	–
M	Asian/Pacific Islander	Advanced	25–39	–	✓	–	✓	–	–
M	Asian/Pacific Islander	Advanced	40–49	✓	✓	✓	✓	–	✓
M	Asian/Pacific Islander	Advanced	50–59	–	✓	✓	✓	–	✓
M	Asian/Pacific Islander	Advanced	60+	–	–	✓	✓	–	✓
F	Black	Advanced	25–39	–	–	–	✓	–	–
F	Black	Advanced	40–49	–	✓	–	–	–	–
M	Black	Advanced	25–39	–	✓	✓	✓	–	✓
M	Black	Advanced	40–49	–	✓	✓	✓	–	✓
M	Black	Advanced	50–59	–	–	✓	✓	✓	✓
F	Other	Advanced	25–39	–	–	–	–	–	✓
F	Other	Advanced	40–49	–	✓	–	✓	✓	✓
F	Other	Advanced	50–59	–	–	✓	✓	✓	✓
M	Other	Advanced	40–49	✓	✓	✓	✓	✓	✓
M	Other	Advanced	50–59	–	✓	✓	✓	–	✓
M	Other	Advanced	60+	–	✓	–	–	–	–
F	White	Advanced	40–49	–	–	–	✓	–	–
F	White	Advanced	50–59	–	✓	✓	✓	✓	✓
M	White	Advanced	25–39	–	✓	✓	✓	✓	✓
M	White	Advanced	40–49	–	–	–	✓	✓	✓
M	White	Advanced	50–59	–	–	✓	✓	✓	✓
M	White	Advanced	60+	–	–	–	–	✓	✓

Table 1. *Results for the CPS work hours dataset: selected groups at levels* $\alpha = 0.01$ *and* 0.05. *The reference group corresponds to Female/White/Some college/25–39.*

Maura John, Markus J Ankenbrand, Carolin Artmann, Jan A Freudenthal, Arthur Korte, and Dominik G Grimm. Efficient permutation-based genome-wide association studies for normal and skewed phenotypic distributions. *Bioinformatics*, 38(Supplement_2):ii5–ii12, 09 2022.

Arun Kumar Kuchibhotla. Exchangeability, conformal prediction, and rank tests. *arXiv preprint arXiv:2005.06095*, 2020.

Yonghoon Lee, Eric Tchetgen Tchetgen, and Edgar Dobriban. Batch predictive inference. *arXiv preprint arXiv:2409.13990*, 2024.

Erich L Lehmann. Parametric versus nonparametrics: two alternative methodologies. In *Selected works of EL Lehmann*, pages 437–445. Springer, 2012.

Erich L Lehmann and Joseph P Romano. *Testing Statistical Hypotheses*. Springer Science & Business Media, 2022.

Erich Leo Lehmann and Howard JM D'Abrera. *Nonparametrics: statistical methods based on ranks*. Springer New York, 2006.

Jackson H Loper, Lihua Lei, William Fithian, and Wesley Tansey. Smoothed nested testing on directed acyclic graphs. *Biometrika*, 109(2):457–471, 2022.

Henry B Mann and Donald R Whitney. On a test of whether one of two random variables is stochastically larger than the other. *The annals of mathematical statistics*, pages 50–60, 1947.

Pierre Neuvial. Asymptotic properties of false discovery rate controlling procedures under independence. *Electronic Journal of Statistics*, 2:1065–1110, 2008. .

Pierre Neuvial. Asymptotic results on adaptive false discovery rate controlling procedures based on kernel estimators. *Journal of Machine Learning Research*, 14:1423–1459, 2013.

Aaditya Ramdas, Nicolás García Trillos, and Marco Cuturi. On wasserstein two-sample testing and related families of nonparametric tests. *Entropy*, 19(2):47, 2017.

Paul R Rosenbaum. An exact distribution-free test comparing two multivariate distributions based on adjacency. *Journal of the Royal Statistical Society Series B: Statistical Methodology*, 67(4):515–530, 2005.

Murray Rosenblatt. Remarks on a multivariate transformation. *The Annals of Mathematical Statistics*, 23(3):470–472, 1952. .

Jarkko Salojärvi, Olli-Pekka Smolander, Kaisa Nieminen, Sitaram Rajaraman, Omid Safronov, Pezhman Safdari, Airi Lamminmäki, Juha Immanen, Tianying Lan, Jaakko Tanskanen, et al. Genome sequencing and population genomic analyses provide insights into the adaptive landscape of silver birch. *Nature genetics*, 49(6):904–912, 2017.

Barbara E Stranger, Alexandra C Nica, Matthew S Forrest, Antigone Dimas, Christine P Bird, Claude Beazley, Catherine E Ingle, Mark Dunning, Paul Flicek, Daphne Koller, et al. Population genomics of human gene expression. *Nature genetics*, 39(10):1217–1224, 2007.

Volker Strassen. The existence of probability measures with given marginals. *The Annals of Mathematical Statistics*, 36(2):423–439, 1965.

[*Received on* ? ? 2025. *Editorial decision on* ? ? 2025]
